# Personality as a Predictor of Time-Activity Budget in Lion-Tailed Macaques (*Macaca silenus*)

**DOI:** 10.3390/ani12121495

**Published:** 2022-06-08

**Authors:** Charlotte E. Kluiver, Jolanda A. de Jong, Jorg J. M. Massen, Debottam Bhattacharjee

**Affiliations:** 1 Animal Behaviour and Cognition, Department of Biology, Utrecht University, Padualaan 8, 3584 CH Utrecht, The Netherlands; c.e.kluiver@uu.nl (C.E.K.); j.j.m.massen@uu.nl (J.J.M.M.); 2 Department of Applied Biology, Aeres University of Applied Sciences, Arboretum West 98, 1325 WB Almere, The Netherlands; jolanda.anneke@gmail.com

**Keywords:** individual variation, persistence, sociability, affiliation, anxiety, food-related behaviour, activity, resting

## Abstract

**Simple Summary:**

Time-activity budgets describe how animals divide their day into various behaviours and activities, e.g., time spent foraging or resting. Activity budgets can serve as crucial indicators of energy intake and expenditure, providing better knowledge of a species’ lifestyle. The conventional trend has been to explore group-level time-activity budgets; however, individuals may also vary in their time-activity budgets (e.g., one individual foraging more than another), with the influencing mechanisms still poorly understood. We propose that animal personality, a behavioural and cognitive profile that makes one individual different from another, may explain why individuals vary in their time-activity budgets. We used a multi-method approach comprised of behavioural observations and experiments to assess the personality traits of lion-tailed macaques. The observed traits were used to predict individual time-activity budgets, broadly categorised into food-related, active, and resting behaviours. We then discuss the significance of this novel approach in light of lion-tailed macaque ecology, conservation, and welfare.

**Abstract:**

Time-activity budget, i.e., how a population or an individual divides their day into various behaviours and activities, is an important ecological aspect. Existing research primarily focused on group-level time-activity budgets, while individual variations have only been reported recently. However, little is known about how consistent inter-individual differences or personalities influence time-activity budgets. We examined the personalities of lion-tailed macaques (*Macaca silenus*) and investigated their influence on individual time-activity budgets. The resulting personality traits, namely persistence, sociability, affiliation, and anxiety, were used to predict the three broad categories of the time-activity budget—food-related, active, and resting behaviours. We found that persistence and sociability positively predicted the time spent being active. Food-related behaviours were positively predicted by persistence, while anxiety was found to influence them negatively. The time spent resting was negatively predicted by persistence. We did not find an effect of affiliation on the time-activity budgets. We discuss these findings in light of the ecology of lion-tailed macaques. Our study highlights the importance of a novel approach that uses animal personality traits as predictors of individual time-activity budgets and offers insights regarding the use of personality assessments in conservation and welfare activities.

## 1. Introduction

For many species, time-activity budgets are used to construct a comprehensive overview of behaviour (e.g., [1,2,3,4,5,6,7,8]). A time-activity budget illustrates the percentages of time spent on various behaviours and activities and provides insights into the relative importance of behaviours concerning energy expenditure and intake [9]. Time-activity budgets often contain behavioural components related to feeding, activity, and resting. These behaviours, and food-related behaviour in particular, regulate the energy available to an individual and are thus crucial to growth, reproduction, and survival. In a number of species, a larger energy budget due to increased food consumption resulted in a higher growth rate [10,11,12,13]. In addition, an increase in dietary energy improved reproduction efficiency in Indonesian Kosta goats in terms of pregnancy length, age of puberty, and litter size [14]. Additionally, longer foraging bouts in breeding Cape gannets (*Morus capensis*) were linked to a higher chick survival rate [12]. 

The existing research focused primarily on group or species-level time-activity budgets (e.g., [15,16,17,18]), while little information exists at the individual level. Variations in individual time-activity budgets have been reported in social species, indicating differences in fitness; however, the underlying mechanisms are still poorly understood (see [19]). Moreover, classic individual characteristics such as sex, age, and dominance rank relationships could not explain variations clearly [16,20]. Here, we propose a novel approach of using consistent inter-individual differences in behaviour or “personalities” as a potential driver of individual time-activity budgets in animals. Investigating individual-level responses can help understand the ecological processes and fitness consequences better than simply representing a “species-typical pattern”. 

Inter-individual behavioural variation has been documented across many taxa, including birds [21], reptiles [22], fish [23], amphibians [24], and mammals [25]. When such behavioural variations are consistent over time and context, they may be ascribed to personalities [26,27]. The five major axes of animal personality include boldness, exploration, aggressiveness, activity, and sociability [27], although there is considerable variation in how many traits animals portray and which behaviours are included within a trait [28]. For example, in primates, some additional traits have been described: i.e., fearfulness, anxiousness, playfulness, and persistence [29].

Animal personalities have considerable evolutionary significance as they influence survival [30,31], reproductive success [31,32], and growth rate [33,34,35]. Moreover, previous studies have generated some evidence regarding the influence of animal personalities on specific components of time-activity budgets. For example, foraging behaviour was found to be impacted by boldness in fallow deer (*Dama dama*) [36], barnacle geese (*Branta leucopsis*) [37], and fish species [38]. Furthermore, links between personalities and resting metabolic rates, i.e., energy spent for resting and activities, have been reported in a number of species [39,40]. Nevertheless, to our knowledge, personality traits have never been applied to predict variations in individual time-activity budgets at an extensive scale. In this study, we assessed the personality traits of lion-tailed macaques (*Macaca silenus*) and used them as predictors of individual time-activity budgets. 

Lion-tailed macaques are a highly relevant species for investigating time-activity budgets due to their endangered and habitat-specialist status. They are distributed across the southwestern region of India (Western Ghats) and have been experiencing intense anthropogenic pressure [41]. The lion-tailed macaque’s habitat is severely fragmented, with its quality continuing to decline [42,43], and these disruptions can be highly challenging for their survival [44,45]. The disturbance caused by anthropogenic activities can alter time-activity budgets [46,47,48,49]. For example, low habitat quality forced Barbary macaques (*Macaca sylvanus*), a species rather closely related to lion-tailed macaques, to spend more time collecting sufficient resources at the cost of resting [17]. Therefore, time-activity budgets may also help us understand the adaptation to changing habitats [9,50].

Dhawale et al. [51] have shown that lion-tailed macaques alter their behaviour depending upon the degree of anthropogenic activities. They found that aggressive behaviours were more often displayed in forest patches cleared of vegetation as compared to the forest interior. In human-dominated habitats, individuals exhibited short grooming duration and low reciprocity, suggesting a disrupted affiliative structure within the group. Furthermore, a decline in the foraging behaviour was reported in habitats with human resources. Interestingly, they found significant inter-individual variations in the behavioural responses, but neither dominance rank nor sex could explain it [51]. Other potential drivers for this variation, such as personality, remain to be tested. Based on behavioural observations of lion-tailed macaques, three personality dimensions are known—(i) extraversion, including affiliative and sociable behaviours, (ii) agonistic, and (iii) curiosity, including bold and cautious behaviours [52].

In this study, we used a multi-method approach comprised of behavioural observations and experiments to determine the personality traits of two captive lion-tailed macaque groups (total *n* = 11). Independent of the personality data, we conducted separate behavioural observations to calculate the time-activity budgets of the individual animals. The time-activity budget was broadly classified into food-related, active, and resting behaviours. Given that animal personalities are individual behavioural and cognitive constructs that make one individual different from another, we hypothesized that it might also influence the time spent on specific behaviours and thus explain the variation in time-activity budgets. Using our combined approach, we expect to gain information on the personality traits of lion-tailed macaques, especially on their explorative and persistent behaviours [53]. As a result of our multi-method approach of observations and experiments, the observed personality traits in our study might differ from the previously reported ones in lion-tailed macaques. Therefore, informed predictions are difficult to make beforehand. However, we predict food-related and activity-related behaviour to be performed more by individuals scoring high on extraversion traits as it has been shown that individuals prone to being sociable and extraverted are highly active [54,55,56]. Furthermore, we predict all three behavioural states to be impacted by agonistic personality types as the energy demands associated with agonistic behaviours may impact food-related behaviours, activity, and resting [57]. For example, aggressive captive water-striders (*Aquarius remiges*) are more active than their non-aggressive counterparts [58]. Similarly, bold sticklebacks (*Gasterosteus aculeatus*) were found to show high levels of activity [59].

## 2. Materials and Methods

### 2.1. Subjects, Study Sites, and Ethical Considerations

This study included two captive lion-tailed macaque groups housed in the Netherlands. The first group (*n* = 8) was housed at the Apenheul Primate Park (hereafter AP) in Apeldoorn. This group consisted of one adult male, five adult females—one of which was lactating—one juvenile male, and one infant male. Individuals from five years old onwards were considered adults, from one to four years old were considered juvenile, and less than one year old were considered as infants [60]. The infant was excluded from this study as he did not move independently from his mother. The second group (*n* = 5) was housed at Blijdorp Zoo (hereafter BZ) in Rotterdam and consisted of two adult males and three adult females. One adult male died before all data were collected. Therefore, he was excluded, resulting in a total sample size of 11 (Table S1).

The AP group had access to an indoor enclosure of approximately 80 m^2^, where they spent the night. The floors were tiled, and no bedding was provided (except for the winter months). The enclosure temperature ranged between 18 and 21 degrees Celsius. When weather conditions were acceptable, access to the indoor enclosure was restricted during daytime, and individuals were only allowed to be in their outside enclosure: an island of 768 m^2^. This area had different vegetation types, including edible plants and large natural trees, thus providing foraging opportunities. There were climbing structures and platforms connected by multiple ropes placed across the island. A mix of vegetables, fruits, seeds, and monkey pellets was thrown onto the island multiple times (at least three times) a day or placed in feeding structures to encourage foraging behaviour. Unlike the inside enclosure, the outside area was visible to visitors. 

In the BZ group, the animals had access to two enclosures. The outdoor island was 100 m^2^ and offered natural trees and vegetation. The indoor area was 106 m^2^. It consisted of a larger section (8.5 m × 10 m) and two small chambers (3 m × 4 m and 2.5 m × 3.5 m) used for feeding and separation whenever required. The floor was covered with woodchips, and enrichment was available in the form of branches and wooden climbing structures. Food was provided three times a day (morning, noon, and evening). The animals were given a balanced and alternating diet consisting of fruits, vegetables, fresh herbs, small insects, eggs, nuts, seeds, and monkey pellets. In addition, they were able to feed on insects and vegetation found on the outside island. Both areas were visible to visitors, except for the feeding and separation chambers inside. Both the AP and BZ groups had ad libitum water availability 24/7. Enclosures are depicted in Figure S1. 

### 2.2. Experimental Design and Data Collection

#### 2.2.1. Time-Activity Budgets

Data were collected using an instantaneous group scan method [61]. Scans were collected from three to five days a week between 0900 and 1600 h. Scans were made throughout the day with a 20 min interval so that they could be performed in between focal observations. Scans lasted for a maximum of three minutes, in which the observer located all individuals, and the behavioural states were noted (Table S2). The behaviours included *autogroom, drink, active forage, food search, passive feeding, move, play, rest, sit, sit-alert, sleep, stand-alert, survey*, and *out of sight.* A total of 3325 scans (AP: 1869; BZ: 1456) were collected from April to July 2021 (AP) and from April to June 2021 (BZ).

#### 2.2.2. Personality Traits

Both observational and experimental data were collected to assess consistent inter-individual differences. Data collection in the AP group was performed by a single observer, while in the BZ group, two observers were present. All the animals were habituated to the observers before data collection. Behaviours were recorded using Canon Legria HF R806 cameras (Canon, Rotterdam, the Netherlands) placed on tripods. Observers moved freely along enclosure boundaries to ensure that the maximum number of individuals remained in the frame. The observers did not interact with the animals during the observations and experiments. 

***Observational approach.*** Data collection took place from April to June 2021 (AP) and from January to February 2021 (BZ). Observations were performed from three to five days a week using a continuous focal sampling (20 min long/focal) method [61]. We used an extensive ethogram (Table S3) to document all behaviours shown by the individuals. No individual was observed on two consecutive occasions, and the sampling order was randomised. Each individual was observed during a morning session (0730–1200 h) and an afternoon session (1300–1700 h). A total of 3780 min of data was collected (average per individual: AP = 360 ± 19 min, BZ = 252 ± 24 min).

***Experimental approach.*** In addition to focal observations, individuals were subjected to four categories of novelty experiments using novel objects, novel foods, predator models, and food puzzles (Figure S2). Each category had two variants, resulting in a total of eight different experiments. These experiments created the conditions for the observation of behaviours related to boldness, anxiety, exploration, and persistence. Such behaviours may be rare and thus challenging to document with the use of focal observations. The importance of the temporal consistency of behaviour related to personality was addressed by repeating all experiments after an interval of 14 (AP) and 17 weeks (BZ). Thus, all animals were subjected to 16 experiments in total.

Novel objects included two types of dog toys—(i) rubber frisbees (Ø 25 cm) and (ii) plastic balls (Ø 15 cm), typically used as food enrichment for dogs—but did not contain any food items.

We used two types of food puzzles—(i) a wooden maze box (~30 cm × 40 cm × 15 cm, secured on tree stems and placed 50 cm above ground level; a plexiglass front with strategic openings that individuals were allowed to insert their fingers into in order to move and retrieve the food items. Walnuts and boiled eggs were used as rewards for the AP and BZ groups, respectively), and (ii) pipes (approximately 70 cm in length, Ø 15 cm with one-sided perforations; pipes were secured 100–200 cm above the ground. Individuals could only retrieve food by rotating and holding the pipes. We used hazelnuts and a mix of nuts, raisins, and sunflower seeds for the AP and BZ groups, respectively). 

We decided on novel foods by considering diet and previous food history (AP: peeled lotus root, cassava root; BZ: pineapple, kiwi). Monopolisation was minimised by providing two identical objects and puzzles and multiple intact or cut pieces of novel food. Predator models possessed visual cues that are known to provoke anti-predator responses in macaques [62,63]. We used a rubber snake with a pattern similar to a reticulated python (approximately 150 cm in length), a stuffed lioness (BZ: ~100 cm), and a plush tiger (AP: ~100 cm in length). In the BZ group, both predator models were placed at an approximate distance of one meter from the enclosure boundaries, out of reach of individuals whilst still being approachable. In AP, the enclosure layout did not permit such a set-up. We placed the models on a bridge (five meters from the island edge), but it did not elicit a response (possibly due to the significant distance between the model and the animals). Therefore, we placed the snake model on the island itself. We chose not to place the tiger on the island due to the risk of the individuals damaging it. Alternatively, we created a platform approximately one and a half meters away from the edge of the island by placing crates in the water, on which the tiger was positioned.

We video-recorded all experimental sessions. Both rounds of experiments lasted for two–three weeks, with one trial per day. The first round was conducted in May 2021 (AP) and February 2021 (BZ), the second round in September 2021 (AP) and June and July 2021 (BZ). The order of the experiments was semi-randomised so that the predator model experiments would not be performed on consecutive days. All the experimental items were placed while the individuals were confined to the adjacent enclosure, and video recording began once the individuals gained access to the experimental setup. We recorded the novel object trials for 60 min. Food puzzle trials lasted until the puzzles were solved, with a maximum length of 60 min. The novel food trials ended when all individuals had had a chance to approach and inspect the food items, and the maximum duration was 60 min. The predator trials were conducted for 30 min. 

### 2.3. Statistical Analysis 

#### 2.3.1. Data Coding and Preparation

Two observers coded all the videos to the nearest second in a frame-by-frame approach using the VLC media player 03.01.14 (VideoLAN, Utrecht, The Netherlands). We used a two-way mixed model to attain an intraclass correlation coefficient (ICC 3,k) based on the mean of both observers and their consistency [64]. Behavioural variables were added to the model as random factors and observers as fixed factors. We obtained high inter-rater reliability scores (ICC (3,k) = 0.98, *p* < 0.001).

***Time-activity budgets.*** Time-activity budgets were measured by the percentage of group scans at which specific behavioural states were observed. Data were corrected for the times spent out of sight. We calculated the occurrence for each behavioural state and divided it by the total number of scans at the group and individual levels. The behavioural state *absent from the troop* was removed due to non-occurrence. *Sit* and *rest* were combined under the label *rest* as it was difficult for observers to differentiate them. To reduce the number of statistical tests (and the accompanying risk of type I errors), the behavioural states were summarized into the following categories: (1) food-related behavioural states (*drink*, *active forage, food search, passive feeding*, *survey*), (2) activity (*move, play, sit-alert, stand, stand-alert, autogroom*), and (3) resting (*sleep, rest*). 

***Personality traits.*** Focal observations resulted in variables of duration behaviours (s/min) and event behaviours (occurrence/minute). Values were calculated per individual. In preparation for further analysis, focal data were separated into two observation phases to test for temporal consistencies of behavioural variables across the entire observation period. We selected the phases so that the observation time for each individual was as similar as possible (AP: 180 ± 19 min/individual/phase; BZ: 126 ± 18 min/individual/phase) while ensuring that the observation dates were consistent across phases and individuals. 

Relevant parameters differed across personality experiments and were coded accordingly (see Table S4 for descriptions). *Proximity* (s/hour) is the time spent within a radius of one meter (novel objects, novel foods, food puzzles) or two meters (predator models) to the experimental items. *Latency to approach* (s) defined the time it took for an individual to reach a one-meter proximity to an experimental item, starting from the moment they entered a five-meter radius. When an individual did not approach, *latency* was scored as NA. Once individuals made contact with the items, *handling* (s/hour; novel objects) or *manipulation* (s/hour; food puzzles) was scored. Eating novel food was represented by *eat* (yes/no). Predator trials also included the variable *approach* (occurrence/hour): the instances when an individual entered a two-meter radius of the predator model from a five-meter radius.

#### 2.3.2. Analysis

We performed all analyses on R version 4.0.1 using the RStudio interface version 1.2.959 [65]. All variables were standardized using *z*-scores for each zoo before being combined. The variable *play* could not be standardised due to its absence in the BZ group. We therefore decided to remove it.

***Time-activity budgets.*** We calculated time-activity budgets for (i) all individuals combined in order to have species-level information, (ii) the AP and BZ groups separately to assess the possible influence of living conditions, and (iii) all individuals separately. 

We compared the three main categories using goodness of fit chi-square tests on a group level. The time-activity budgets between the AP and BZ groups were compared using a generalised linear model (GLM). We used the number of occurrences of a behavioural category as the response variable (rather than the proportion data used previously) and the zoo (or group) as a fixed effect. A Poisson error distribution with a “log” link function was used in the model. We added the number of scans as an offset (log-transformed). Moreover, we examined the individual variation in behavioural states using goodness of fit chi-square tests. Analyses were also run without the lactating female in the sample since lactation may impact the time-activity budget, favouring resting over foraging as an energy-saving strategy [15]. We assessed whether potential differences in the time-activity budget were due to individual variation rather than differences in reproductive state. 

***Personality traits.*** Behaviours reflecting animal personality should be consistent over time (i.e., repeatable). The temporal consistency of collected behavioural variables was assessed using a two-way mixed-model intraclass correlation (ICC (3,1)) [53] that compared the first and second rounds of experiments with phases one and two of the observations. Variables with ICC values ≥ 0.3 and *p* < 0.05 were retained for further analysis. In order to avoid bias from variables with low occurrences, we dropped variables where over half of the individuals had zero values. This resulted in the removal of all predator model variables as most individuals did not engage. In addition, we removed the variables *eat*, *travel together, object play, o-mouth, lunge passive, hang, fall, embrace passive,* and *avoid passive.*

Apart from temporal consistency, we also checked the contextual consistency of the experiments by calculating Cronbach’s alpha. This value represents the similarity of behaviours shown in various situations. A value of >0.7 is considered sufficient [66] and signifies that the behavioural response to different experiments is comparable, indicating that behaviours are consistent over context.

Once the repeated variables were finalized, a principal component analysis (PCA) was conducted following the standardized approach, as performed in [53]. However, we first calculated the average values for each repeatable variable from the observation and experiments. All assumptions for PCA were met. The number of principal components to select was based on the inspection of a generated scree plot using an unrotated PCA, the amount of variance explained by components (>70%) and eigenvalues (>1) [67]. To retain statistical power from the PCA, we decided on a strict approach for accepting variables. We increased the required communality score from 20% (as in [53]) to 70%, as advised for small sample sizes [68], which led to the removal of *proximity of balls, latency to balls,* and *look around*. In addition, we used a Varimax rotation and considered factor loadings of >±0.5 as salient [67]. Furthermore, all variables loading onto multiple components were removed following our strict acceptance policy, which resulted in the exclusion of *approach passive, proximity,* and *travel.* Components were labelled as personality traits based on the nature of the loaded behavioural variables. The possible effects of age and sex on personality components were considered by running linear mixed-effect models (LMM). Separate models were run for each personality component. The personality components were entered as the response variable. Age (ranging from 1 to 29) and sex (*n*_males_ = 3; *n*_females_ = 8) were fixed effects. Location (*n*_AP_ = 7; *n*_BZ_ = 4) was entered as a random effect. Model diagnostics were conducted (residual distribution and dispersion).

In addition to factor loadings, we extracted factor scores. These values represent the contribution of each individual to the different components; in other words, how much an individual relates to a personality trait. Factor scores will therefore be referred to as *personality scores*. 

***Personality as a predictor of time-activity budgets.*** To see whether found personality traits could predict individual time-activity budgets, we ran multiple generalised linear mixed effect models (GLMM). Separate models were run for the following response variables—(1) food-related, (2) activity, and (3) resting. The data consisted of count variables; therefore, we used a Poisson error distribution with a “log” link function. We added the number of scans (log-transformed) as an offset variable to correct differences in the number of scans per individual. Personality traits with corresponding scores were entered as fixed effects, and location was entered as a random effect. Model fit was assessed by inspecting residual distribution, dispersion, and the AIC values. The models with the lowest AIC were retained. Null vs. full model comparisons were run. 

***Age and sex as predictors of time-activity budgets.*** Apart from personality, the effects of age and sex on the different behavioural states were assessed separately using GLMMs. Response variables included (1) food-related, (2) activity, and (3) resting behaviours. Age and sex were entered as fixed effects, while the different locations were added as a random effect. A Poisson error distribution with a “log” link function was used. We added the number of scans as an offset variable (log-transformed). We conducted model diagnostics (residual distribution and dispersion). When residual dispersion was significantly high, we used a negative binomial family rather than a Poisson family. We performed null versus full model comparisons using likelihood ratio tests. 

***Statistical packages.*** GLMs were run with the “glm” function from the “stats” package [65]. For GLMMs, we used the “glmer” function from the “GlmmTMB” package [69]. Model residuals were inspected using the “testResiduals” and “simulateResiduals” functions from the “DHARMa” package [70]. Null versus full model comparisons were made with the “lrtest” function from the “lmtest” package [71]. The goodness of fit tests were run using the “prop.test” function of the “stats” package [65]. ICCs were run using the “ICC” function from the “psych” package [72]. When variables contained missing data (e.g., an experimental item was not approached, resulting in NA for latency), the “iccNA” function from the “iirNA” package was used [73]. PCA was performed with the “full_factor” function of the “Radiant.multivariate” package [74]. LMMs were conducted with the “lmer” function from the “lme4” package [75] and the “afex” package [76]. 

## 3. Results

### 3.1. Time-Activity Budgets

**Figure 1:**
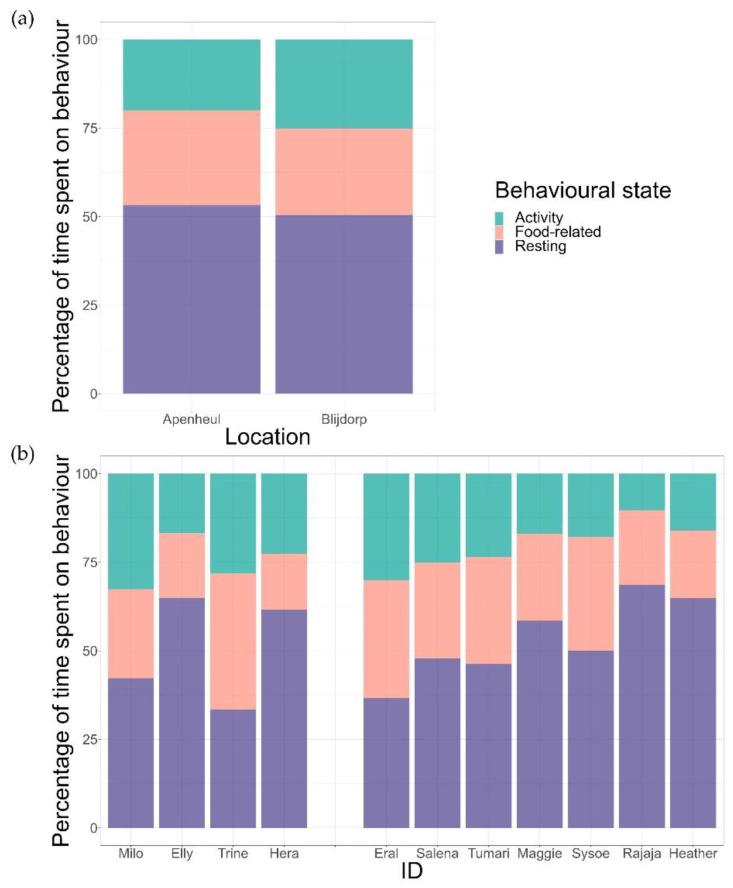
(**a**) Time-activity budget of AP and BZ groups; (**b**) Time-activity budgets of all individuals separated by location, with BZ on the left and AP on the right.

We found that the individuals spent 5.9% ± 4.9% of the scans out of sight. All scans containing this variable were removed prior to continuing with further analyses. Inspection of group-level time-activity budgets showed that the time spent on behavioural states differed significantly between states (goodness of fit, χ^2^ = 750.88, df = 2, *p* < 0.001). Individuals spent the majority of their day resting (49 ± 11%), followed by food-related behavioural states (24 ± 7%) and activity (21 ± 7%). There was no significant difference between the AP and the BZ groups (GLM, *z* = 1.55, *p* = 0.122) (Figure 1a). However, we found variations in time-activity budgets at the individual level (food-related behaviour: goodness of fit, χ^2^ = 83.02, df = 10, *p* < 0.001; activity: χ^2^ = 77.70, df = 10, *p* < 0.001; resting: χ^2^ = 176.39, df = 10 *p* < 0.001) (Figure 1b). Overall, we found that food-related behaviour ranged between 16% and 38%, activity varied from 16% to 33%, and resting from 33% to 69% (see Table S5 for data on all separate behavioural states). Analyses without the lactating female in the sample were comparable (results are presented in the Supplementary Materials); we therefore decided to keep our initial sample size intact for further analyses. 

### 3.2. Personality Traits

Inter-rater reliability was deemed sufficient (ICC (3,k) = 0.988, *p* < 0.001). The ICC analyses yielded 26 repeatable variables. ICC values ranged from 0.0 to 0.98 (Table 1).

After eliminating variables with low communality scores (<0.7), 23 repeatable variables were obtained and used for the PCA. Final communality scores ranged between 76% and 98%, indicating that variance is sufficiently explained by the extracted principal components (PCs). We extracted 4 PCs that explain 90% of the total variance. The first PC explained 30% of the variance and had high positive loadings (>0.5) of manipulation of the frisbees, balls, boxes, and pipes (+), as well as salient negative loadings of *leave passive* (−) and *pass by passive* (−). As the majority of variables represent a continuous manipulation of the experimental items, we named this PC “persistence”. The second PC explained 24% of the variance and contained the behaviours *approach* (+), *climb* (+), *follow* (+), and *pass by* (+). Due to the nature of these variables, we labelled it as “sociability”. The third component explained 24% of the variance, with the behaviours *body shake* (+), *contact sit* (+), *follow passive* (+), and *groom* (+). We labelled this PC as “affiliation”. The last PC explained 13% of the variance and included *autogroom* (+) and *scratch* (+) and was therefore labelled as “anxiety”. Behavioural variables with salient factor loading scores for multiple components, i.e., cross-loadings, were excluded (Table 2). The component persistence was the only component with experimental variables. These manipulation variables showed contextual consistency (Cronbach’s alpha: 0.96).

**Figure 2:**
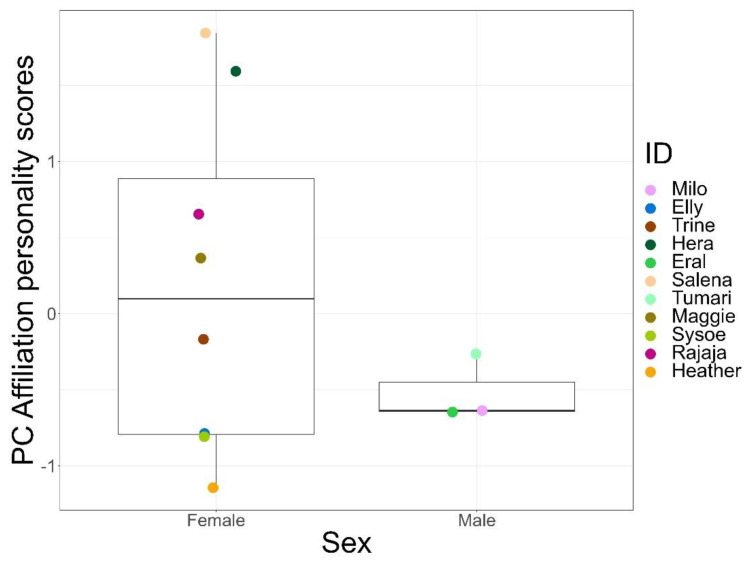
Personality scores for the affiliation component of females and males. Different coloured dots indicate individuals.

Residuals of the models used to assess the effects of sex and age on the personality components met all assumptions. Dispersion tests and Kolmogorov–Smirnov tests were not significant for the models. We did not find any effects of sex or age on the persistence, sociability, and anxiety components (Table S6). However, sex significantly predicted the personality scores of the affiliation component (LMM, df = 8, t = −2.44, *p* = 0.04; Figure 2), with females scoring higher on this trait than males. 

Individual personality scores are given in Table 3.

### 3.3. Personality as a Predictor of Time-Activity Budget

Residuals of the models used to assess personality as a predictor of time-activity budget met the assumption of normal distribution. One-sample Kolmogorov–Smirnov and dispersion tests were not significant (Table S7). Model fit was significantly improved by including all fixed effects (persistence, sociability, affiliation, and anxiety) for food-related behavioural states (LRT, df = 6, χ^2^ = 13.36, *p* = 0.004) and activity (LRT, df = 6, χ^2^ = 11.60, *p* = 0.009). Including all fixed effects did not improve model fit for resting; however, the full model had an AIC value identical to the null model; therefore, we decided to continue with the full model.

**Figure 3:**
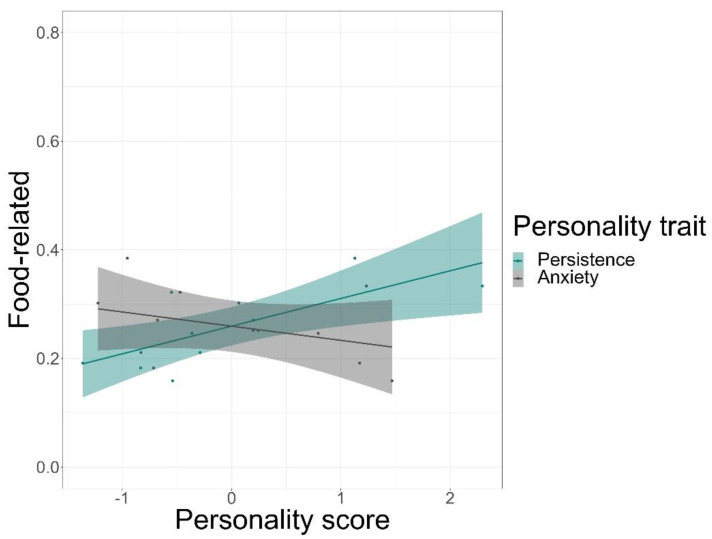
Time spent on food-related behaviours in percentages and its relation to the personality scores of the traits of persistence and anxiety. The solid line is a trend line surrounded by the 95% confidence interval.

**Figure 4:**
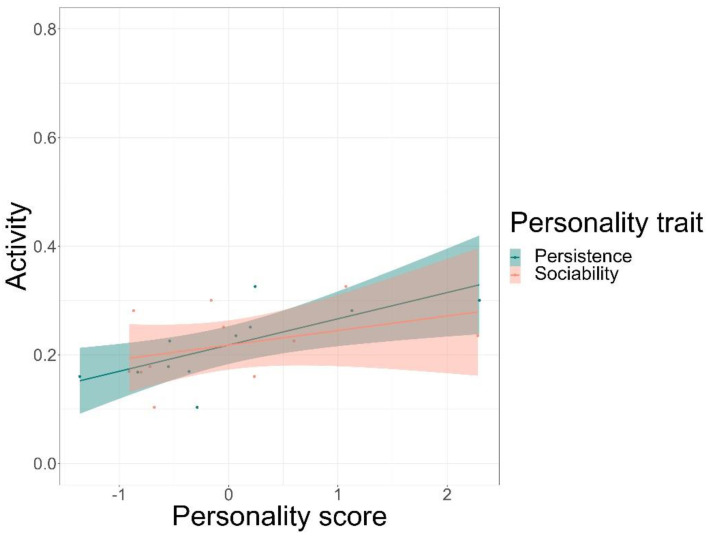
Time spent being active in percentages and its relation to the personality scores of the traits of persistence and sociability. The solid line is a trend line surrounded by the 95% confidence interval.

**Figure 5:**
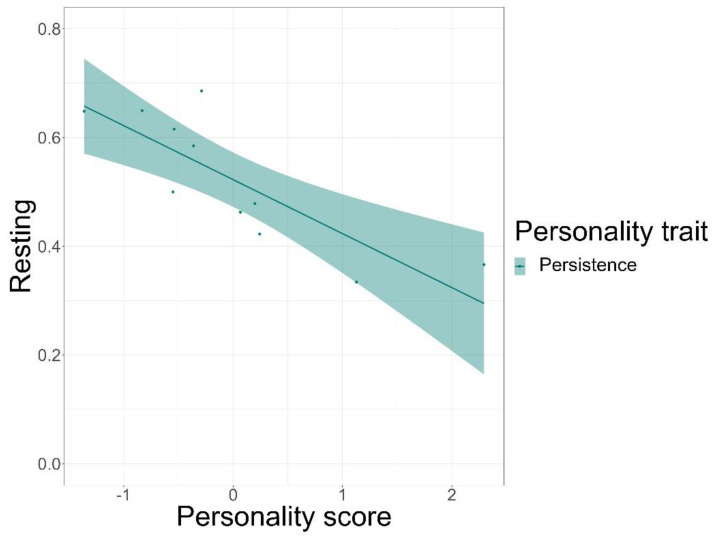
Time spent resting in percentages and its relation to the personality scores of the trait of persistence. The solid line is a trend line surrounded by the 95% confidence interval.

We found that time spent on food-related behaviour was positively predicted by the personality trait of persistence (GLMM, *z* = 5.72, *p* < 0.001) and negatively by anxiety (GLMM, *z* = −3.20, *p* = 0.001), but not by sociability or affiliation (Figure 3, see Table S8 for complete test statistics). Time spent being active was positively predicted by persistence (GLMM, *z* = 5.5, *p* < 0.001) and sociability (GLMM, *z* = 3.38, *p* < 0.001), but not by affiliation or anxiety (Figure 4). Finally, persistence (GLMM, *z* = −7.41, *p* < 0.001) was found to negatively predict resting time (Figure 5).

### 3.4. Age and Sex as Predictors of Time-Activity Budgets

**Figure 6:**
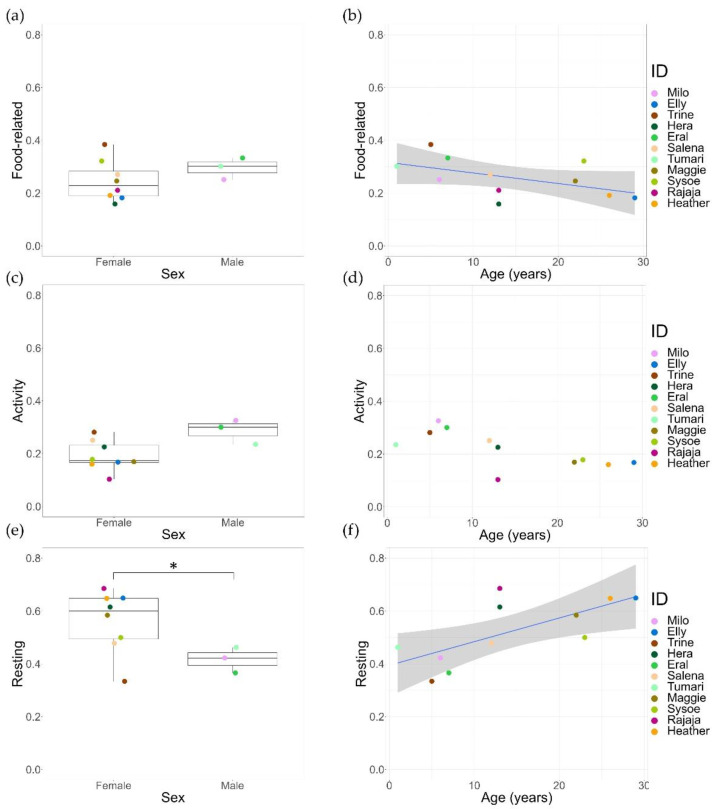
An overview of time spent on specific behavioural states (in percentages) and their relation to age and sex. Solid lines are trendlines, and 95% confidence intervals are given. (**a**) No difference was found in time spent on food-related behaviour between males and females. (**b**) Age is negatively correlated with time spent on food-related behaviours. (**c**) No difference was found between activity levels of females and males. (**d**) No correlation could be found between age and activity. (**e**) Females spent more time resting compared to males, significance indicated with *. (**f**) Age is positively correlated with resting time.

The model fit of activity was improved by including both fixed effects (age and sex) (LRT: df = 1, χ^2^ = 3.94, *p* = 0.047). For food-related behaviours and resting, the model fit was not improved, and the AIC values of the full model were higher (food-related: 116.5; resting: 124.9) compared to the null model (food-related: 115.2; resting: 124.2). Consequently, age and sex were tested separately (see Table S7 for selected models). Time spent on food-related behaviour was negatively correlated with age (GLMM, *z* = −2.06, *p* = 0.036) but showed no difference for sex. Furthermore, activity was not influenced by either age or sex. Finally, females were found to rest more than males (GLMM, *z* = −2.26, *p* = 0.024), and resting time was positively correlated with age (GLMM, *z* = 3.13, *p* = 0.002) (Figure 6, see Table S9 for complete test statistics).

## 4. Discussion

Our findings indicate that individual lion-tailed macaques can vary in time-activity budgets despite similar conditions in captivity. As hypothesised, personality traits were found to be significant predictors of the individual variations in time-activity budgets. Except for affiliation, all other personality traits predicted the different components of time-activity budgets. The time spent on food-related behaviour was predicted positively by persistence and negatively by anxiety. The time spent being active was predicted positively by persistence and sociability, while resting time was predicted negatively by persistence.

We found resting (49%) to be the most prevalent behavioural state, followed by food-related behaviours (24%) and activity (21%). This is not in line with previous research; Kurup and Kumar [1] found that wild lion-tailed macaques inhabiting a large, protected forest spent more than 50% of the day on food-related behaviours, approximately 33% on resting, and 15% on moving. In disturbed forest fragments, it was found that lion-tailed macaques spent 42% of their time foraging and feeding, 16% on resting, and 34% on moving [77]. Contrary to reports on wild populations, the captive groups spent most of their day inactive in this study, most likely due to differences in diet and available resources. Prior research reported similar differences in other species, e.g., wild chimpanzees (*Pan troglodytes*) spent more time “collecting foraging”, which consists of directly eating food items collected from the environment, than their captive counterparts [16]. In the wild, individuals are expected to spend more time seeking food and exhibiting foraging behaviour, while captive conditions offer calorie-dense food multiple times a day, thus reducing the necessity to forage for longer durations. 

In several primate species, such a difference in time-activity budgets between wild and captive groups has often been considered an indicator of the negative welfare of the animals living in captivity [78,79,80,81]. However, using such comparisons to assess welfare can be complex and unjustified (see [82] for a full review); therefore, we cannot make direct welfare assumptions based solely on the observed time-activity budgets within our study.

We did not find a difference in time-activity budget between the Apenheul and Blijdorp groups. Time-activity budget differences among wild lion-tailed macaque groups were accounted for by seasonal variation, group size, and habitat quality; this was also evident in other primate species (i.e., Assamese macaques (*Macaca assamensis*) [83], vervet monkeys (*Chlorocebus pygerythrus*) [20], and chimpanzees [84]. In captivity, however, food items do not depend on seasonality. Likewise, habitat quality may have been similar as both enclosures offered climbing and foraging opportunities with sufficient outdoor space.

We found a significant variation in time-activity budgets at the individual level. Age and sex could not explain all variations. It is noteworthy that the sex ratio in this study was skewed, with only three males as compared to eight females. Moreover, considering such variation within the female group, these findings only highlight a weak link between time spent resting and sex. Additionally, we found that the only lactating female (Rajaja) spent most of the time resting. This is in line with previous studies that showed lactating females rest more at the cost of foraging and feeding as an energy-saving strategy [15,85]. However, our study only contained a single lactating female, and removing her from the sample did not significantly affect the time-activity budget, therefore conclusions should be taken with caution.

The first personality trait of persistence, while it has been reported in other primate species [53,86,87], to our knowledge, it has never been found in lion-tailed macaques. Similarly, the behaviours that loaded on to the persistence trait have not been previously described in relation to lion-tailed macaques’ personality (see [52]). The inclusion of experiments in our study uncovered this trait, demonstrating the added value of our multi-method approach. 

We found two traits related to social behaviour: sociability and affiliation. Sociability contained the behaviours *approach, climb, follow*, and *pass by*. *Climb* may appear to be misplaced in this component. However, we suggest that its loading on this component might be due to the limited space on climbing structures and branches, forcing individuals to move towards and pass by group members frequently. Sociability is thought to be one of the five major axes of personality [27] and has been found in many species [88]. However, our sociability trait indicates an individual’s willingness to associate with others. Nevertheless, it does not contain information about the nature of these interactions, whereas this is described in the affiliation trait. The affiliation trait included *contact-sit* and *groom*, which are behaviours important for social bonding in primates [89,90]. Surprisingly, *body shake*, which is unrelated to affiliation, was found to be part of this component. The weather conditions could explain this; during observations, high levels of rain often led individuals to exhibit this behaviour. Rouff et al. [52] previously reported a lion-tailed macaque personality trait labelled extraversion. Although different in name, it contained sociable and affiliative behaviours similar to the social traits found in this study. In the analysis, they used behavioural variables such as *sociable*, *affiliative*, *solitary*, and *aggressive*, which lack details regarding the specific behaviours shown. It is possible that due to our extensive ethogram and detailed behavioural variables, we could make a distinction between affiliation and sociability. 

Personality research typically describes inter-individual differences in behaviour, irrespective of sex. We found that sex significantly predicted the personality scores of the affiliation component, with females scoring higher than males. However, a relatively new area of research has focused on sex-specific personality, which is based on the consideration that males and females are exposed to different selection pressures throughout their lives, leading to the divergence of behaviour [56]. Sex-specific personality has been reported in several species such as mosquitofish (*Gambusia affinis* and *Gambusia holbrooki*) [91], rock pool prawns (*Palaemon elegans*) [92], zebra finches (*Taeniopygia gutata*) [93], and snow leopards (*Uncia uncia*) [94]. It has also been reported in primates such as barbary macaques (*Macaca sylvanus*), where the traits excitability and tactility showed intersexual variation [95]. Therefore, we believe it is well-justified to consider the affiliation component as a personality trait despite the sex difference, especially since considerable variation was visible among all females (Figure 2). 

Our results indicate that female lion-tailed macaques are inclined to engage in affiliative behaviours that develop and strengthen social bonds within the group. For males, who typically disperse from their natal group, such bonds may be of less importance [96]. This result coincides well with previous macaque studies that showed females performing more affiliative behaviours than males. This was found for lion-tailed macaques [52,97] as well as other macaque species, including rhesus macaques (*Macaca mulatta*), pigtail macaques (*macaca nemestrina*), and long-tailed macaques (*Macaca fascicularis*) [97].

The final trait, anxiety, contained the variables *scratch* and *autogroom*, well-known displacement behaviours in primates that are often related to stress and anxiety [98]. A similar trait containing self-directed behaviours was documented in wild crested macaques (*Macaca nigra*) [99]. A seemingly comparable trait, labelled as anxiousness, has also been reported in other primate species [29]. However, despite the similar name, anxiousness appears to be different from the trait we labelled as anxiety. Individuals with the trait of anxiousness are thought to be hesitant, indecisive, and fearful [29,100]. Our findings do not indicate this, probably because we had to remove all behavioural variables obtained through the predator experiments as most individuals did not approach the predator models, so participation was considered lacking. Therefore, we could not create the circumstances to observe anxiousness in lion-tailed macaque personalities. Nevertheless, we present four personality traits in lion-tailed macaques using a multi-method approach comprised of behavioural observations and ecologically relevant experiments. 

This study finds strong support for the use of personality traits as suitable predictors of all components of the lion-tailed macaque’s time-activity budgets. Persistence was found to positively predict the time spent on food-related behaviour. This suggests that persistent individuals spend more time foraging and feeding than individuals that scored lower on that personality trait. Although natural vegetation, insects, and other small animals such as frogs were freely available on the outdoor islands, individuals still might have needed to be persistent to obtain them. Furthermore, spending time on foraging for these resources even when sufficient food is offered indicates that these individuals are persistent in searching for food. Interestingly, we found that anxiety negatively predicted the time spent on food-related behaviour. Individuals who are predisposed to high-stress levels (see [101]) may be more alert and might prioritise their safety (i.e., to avoid intra and intergroup conflict) over searching for “additional” food. However, there are contrasting reports regarding the relationship between anxiety and food-related behaviour. In line with our results, multiple studies on rats and cattle breeds showed that increased stress levels reduced food intake [102,103]. In contrast, rhesus macaques (*Macaca mulatta*) were shown to have higher caloric consumption in moments of stress [104]. Unfortunately, our study does not allow us to make conclusions regarding the actual intake of food or caloric consumption as we did not record the specific types of food being eaten. Nevertheless, it would be beneficial to include such variables in future research. 

Furthermore, we found that individuals scoring high on the persistence trait spent more time on activity-related behaviour as compared to individuals with lower scores. It is suggestive of the fact that individuals who are inclined to be persistent are generally more active. This is unsurprising as the personality trait of persistence was previously linked to active behaviours such as exploration, tool use, and problem-solving [53,105]. Similarly, persistent individuals are also thought to rest less, which might indicate that the increased activity and food-related behaviours come at the expense of the resting behaviours. Personality has previously been linked to differences in metabolic rates, which may cause individuals to differ in their energy intake and expenditure requirements [106,107]. Perhaps persistent individuals require lower resting times than less persistent individuals, which leads to differential fitness outcomes in animals. This is yet to be explored experimentally. 

Besides persistence, sociability predicted activity-related behaviours positively. These results resemble those previously found in other species; individuals who are prone to being sociable and extraverted had higher levels of activity [54,55,56]. It is well-established in non-human primates that social behaviours can have considerable fitness and survival benefits [108,109,110], suggesting the importance of traits such as affiliation and sociability. For example, in rhesus macaques, maintaining social connectivity within the group was found to increase the relative probability of survival compared to individuals who were less “well-connected” [111].

Time-activity budgets may reflect the adaptation to changing habitats due to anthropogenic activities [9]. Our findings on the variation in individual time-activity budget imply that individuals might differ in their behavioural responses to cope with such changes as personality is known to effectively impact stress response, movement, and dispersion behaviours as well as habitat selection (see [112] for a full review). Human-modified habitats have previously been found to favour specific personality types. For example, female ground beetles (*Carabus convexus*) living in urban areas were shown to be more explorative and risk-taking compared to their rural conspecifics [44]. Additionally, great tits (*Parus major*) from urban areas are thought to have a more proactive personality type than their rural counterparts. This was represented in their different coping strategies when distressed, indicated by a higher pecking rate and more fear screams from urban individuals than rural individuals [113]. Proactive individuals who are inclined to explore and take risks might therefore have high survival values when habitats are disturbed. 

Our findings go beyond previous reports and indicate that persistent individuals are also intrinsically motivated and inclined to spend time searching for food, suggesting their potential to be successful in adverse conditions. This, and the notion they may require less resting time compared to low-persistent individuals, may possibly make them better equipped to cope with the effects of anthropogenic disturbances in their habitat. On the contrary, individuals who are prone to anxiety may be limited in achieving successful adaption as they may find it more difficult to acquire sufficient resources due to their tendency to spend less time on food-related behaviours. This information could also be considered during ex situ conservation efforts such as translocations and reintroductions when deciding which individuals would have the highest post-reintroduction survival potential [24], as prior research has shown that personality affects reintroduction success. For example, explorative zoo-hatched Blanding’s turtles (*Emydoidea blandingii*) were more likely to survive compared to less explorative conspecifics after reintroduction [114]. 

The current study, besides its conservation implications, may also help promote the welfare of captive animals. Personality may predict how an individual will respond to certain situations and should be considered when designing enclosures and husbandry procedures. In the case of our study groups, we would suggest that persistent individuals might prefer enrichment in the form of novel objects or puzzles and would benefit from more food being spread throughout the enclosure to facilitate the need for extensive foraging behaviour, especially when considering the enormous gap between the time spent on food-related activity in the wild and in captivity. In addition, based on personality traits, individuals may handle visitor presence differently. A study on captive Diana monkeys (*Cercopithecus diana*) showed that individuals scoring high on traits such as solitary, aggressiveness, and irritability performed frequent abnormal behaviours when visitor presence was high. In contrast, active, playful, and excitable individuals exhibited more species-typical behaviour such as affiliative behaviours and play [115]. In light of this, we imagine that anxious individuals within our study might benefit from additional hiding places in order to manage visitor presence. This demonstrates how inferences can be drawn from personality traits and associated behaviours regarding what individuals might require for a successful adaptation to captivity, thus allowing for better welfare [116].

## 5. Conclusions

We found individual variations in the time spent on food-related behaviour, activity, and resting. Besides the influences of age and sex, variation could be explained by differences in personality. Our multi-method approach proved effective in describing lion-tailed macaque personality, resulting in the traits of persistence, sociability, affiliation, and anxiety. We provided evidence that all but affiliation are predictors of individual time-activity budget variation. 

Considering the constant changes in natural habitats due to increased anthropogenic activities, understanding the inter-individual differences in the ability of individuals to adapt to novel environmental conditions is crucial when implementing conservation and welfare measures. High-persistent individuals are intrinsically inclined to spending more time searching for food and being more active while resting less. This may make them better equipped to handle unfavourable conditions than high-anxious individuals, who do not have the predisposition for long foraging times. These traits should also be considered during husbandry procedures and enclosure designs. Enrichment preferences may differ based on personality traits; high-persistent individuals may prefer enrichment in the form of novel objects and food puzzles. Furthermore, personality traits may influence how individuals cope with captivity; the welfare of high-anxious individuals may require sufficient hiding places in order to manage visitor presence. This study adds to the growing body of literature stating that variation in time-activity budgets and animal personality are important aspects to consider during conservation and welfare efforts. 

## Figures and Tables

**Table 1:** Temporal consistency of all variables with the test statistics of the intraclass correlation (ICC 3,1) analysis.

Variable	ICC (3,1)	*p*-Value	F-Value	95% CI Upper, Lower
Latency frisbee	0.00	0.50	1.00	−0.50, 0.50
Proximity frisbee	0.06	0.42	1.13	−0.45, 0.54
**Manipulation frisbee**	**0.77**	**0.00**	7.69	0.44, 0.92
Latency ball *	**0.80**	**0.00**	8.99	0.50, 0.93
Proximity ball *	**0.77**	**0.00**	7.80	0.45, 0.92
**Manipulation ball**	**0.98**	**0.00**	126.5	0.95, 0.99
Latency food 1	0.33	0.14	2.01	−0.20, 0.71
Latency food 2	0.23	0.24	1.60	−0.30, 0.65
Latency box	0.12	0.35	1.28	−0.40, 0.58
**Proximity box**	**0.79**	**0.00**	8.34	0.47, 0.92
**Manipulation box**	**0.96**	**0.00**	48.81	0.88, 0.99
Latency pipe	0.00	0.50	1.00	−0.50, 0.50
Proximity pipe	0.10	0.38	1.22	−0.42, 0.57
**Manipulation pipe**	**0.80**	**0.00**	8.94	0.50, 0.93
Approach passive **	**0.94**	**0.00**	33.14	0.84, 0.98
**Approach**	**0.90**	**0.00**	19.04	0.73, 0.97
Attention	0.30	0.17	1.86	−0.23, 0.69
**Autogroom**	**0.97**	**0.00**	58.85	0.90, 0.99
Bipedal stand	0.20	0.26	1.51	−0.33, 0.64
**Body shake**	**0.74**	**0.00**	6.77	0.39, 0.91
Change position	0.00	0.50	1.00	−0.50, 0.50
**Climb**	**0.94**	**0.00**	34.69	0.84, 0.98
**Contact sit**	**0.71**	**0.01**	5.84	0.32, 0.89
**Displace passive**	**0.70**	**0.01**	5.60	0.31, 0.89
**Displace**	**0.73**	**0.00**	6.51	0.37, 0.90
**Follow passive**	**0.88**	**0.00**	15.29	0.67, 0.96
**Follow**	**0.74**	**0.00**	6.56	0.38, 0.90
Forage	0.09	0.39	1.20	−0.43, 0.56
Grab	0.07	0.41	1.15	−0.44, 0.55
Groom passive	0.16	0.31	1.38	−0.37, 0.61
**Groom**	**0.83**	**0.00**	10.66	0.56, 0.94
**Leave passive**	**0.72**	**0.00**	6.05	0.34, 0.89
Leave	0.00	0.50	1.00	−0.50, 0.50
Lie down	0.39	0.11	2.27	−0.14, 0.74
Look around *	**0.96**	**0.00**	44.57	0.87, 0.99
**Pass by passive**	**0.73**	**0.00**	6.38	0.36, 0.90
**Pass by**	**0.92**	**0.00**	23.06	0.77, 0.97
**Play**	**0.82**	**0.01**	NA	0.23, 0.97
Proximity **	**0.87**	**0.00**	14.33	0.66, 0.95
Regurgitation	0.00	0.50	1.00	−0.50, 0.50
**Scratch**	**0.95**	**0.00**	41.78	0.87, 0.98
Sit	0.20	0.27	1.50	−0.33, 0.63
Stand	0.28	0.19	1.78	−0.25, 0.68
Travel **	**0.67**	**0.01**	4.99	0.25, 0.87

Repeatable variables (ICC (3,1) ≥ 0.3, *p* < 0.05) indicated with a bold typeface are retained for further analyses, except for: * Insufficient communality score (<0.7), ** Loads on multiple principal components during PCA (see later).

**Table 2:** Variable loadings in the principal component analysis.

Variable	Persistence	Sociability	Affiliation	Anxiety	Communality *h*^2^
Manipulation frisbee	**0.90**	−0.14	−0.23	0.07	88.19%
Manipulation ball	**0.95**	0.04	−0.18	0.16	96.97%
Manipulation box	**0.94**	−0.19	−0.23	0.09	97.35%
Manipulation pipe	**0.95**	0.10	−0.22	0.09	97.26%
Approach passive *	**−0.60**	−0.04	**0.60**	0.27	79.28%
Approach	−0.11	**0.96**	−0.01	0.15	94.63%
Autogroom	0.21	−0.14	0.34	**0.77**	76.27%
Body shake	−0.33	−0.14	**0.82**	0.30	89.17%
Climb	0.31	**0.91**	0.08	0.03	93.49%
Contact sit	−0.35	0.24	**0.85**	−0.19	93.39%
Follow passive	−0.11	0.21	**0.90**	−0.07	87.63%
Follow	−0.06	**0.99**	0.02	0.04	98.41%
Groom	−0.26	−0.24	**0.92**	0.11	98.44%
Leave passive	**−0.72**	0.36	0.00	0.48	88.04%
Pass by passive	**−0.70**	−0.03	0.33	0.46	81.09%
Pass by	−0.22	**0.95**	−0.08	0.01	95.95%
Proximity *	−0.44	**0.61**	**0.60**	−0.15	94.56%
Scratch	0.04	0.18	−0.14	**0.90**	85.89%
Travel *	−0.32	0.40	**−0.52**	**0.54**	83.13%
Eigen value	5.69	4.58	4.49	2.43	
% of variance explained	30%	24%	24%	13%	

Factor loadings > ±0.5 are indicated with a bold typeface, including both positive and negative loadings. * Variable excluded from the component due to multiple high loadings, i.e., cross-loadings.

**Table 3:** Personality scores of all individuals for the persistence, sociability, affiliation, and anxiety components.

ID	Location	Persistence	Sociability	Affiliation	Anxiety
Milo	BZ	0.24	1.07	−0.64	0.20
Elly	BZ	−0.83	−0.80	−0.78	−0.71
Trine	BZ	1.13	−0.87	−0.17	−0.95
Hera	BZ	−0.54	0.60	1.59	1.47
Eral	AP	2.29	−0.16	−0.65	1.23
Salena	AP	0.20	−0.05	1.84	−0.68
Tumari	AP	0.07	2.28	−0.26	−1.22
Maggie	AP	−0.36	−0.91	0.37	0.79
Sysoe	AP	−0.55	−0.72	−0.81	−0.47
Rajaja	AP	−0.29	−0.68	0.66	−0.82
Heather	AP	−1.36	0.24	−1.14	1.17

## Data Availability

The data presented in the study are available in the supplementary files. Supplementary File S1: Datasets. Supplementary File S2: Rscripts.

## Supplementary Materials

The following supporting information can be downloaded at: https://www.mdpi.com/article/10.3390/ani12121495/s1. Supplementary file S1: datasets. Supplementary file S2: Rscripts. Table S1: Description of study animals, including individual ID, housing location, date of birth, sex, parents, and rearing conditions. Figure S1: (a) Outdoor island at Apenheul Primate Park, Apeldoorn. Individuals were not observed inside. (b) Outdoor island at Blijdorp zoo, Rotterdam. (c) Indoor enclosure at Blijdorp zoo, Rotterdam. Table S2: Behavioural states used to construct time-activity budgets (Adapted from Dhawale et al., 2020). Table S3: Ethogram containing behaviours related to general activity, social and sexual interactions, dominance and submission, conflicts and aggression, and tension. Figure S2: Personality experiments. (a) Food puzzle: puzzle pipes; (b) Food puzzle: puzzle boxes; (c) Novel food: kiwi; (d) Novel food: pineapple; (e) Novel object: plastic balls; (f) Novel object: rubber frisbee; (g) Predator model: Lioness; (h) Predator model: rubber python. Table S4: Descriptions of all measures used during novelty experiments. Table S5: Percentage of time spent on specific behavioural states (%) per individual. Table S6: Test statistics for linearised mixed effect models testing the influence of age and sex on the multiple components. Significant results are indicated with a bold typeface. Table S7: Selected models for the generalised linear mixed-effect analysis with the behaviour of interests, fixed effects, random effects, and the corresponding one-sample Kolmogorov–Smirnov test output. Table S8: Test statistics of the generalised linear mixed effect models, with behavioural states as response variables, and the personality traits of persistence, sociability, affiliation, and anxiety as fixed effects while controlling for location. Significant results are indicated with a bold typeface. Table S9: Test statistics for generalised linear mixed effect models testing the influence of age and sex on the multiple components. Significant results are indicated with a bold typeface.

## References

[1] Kurup G.U., Kumar A. (1993). Time Budget and Activity Patterns of the Lion-Tailed Macaque (*Macaca silenus*). Int. J. Primatol..

[2] Rehman E.U. (2022). Documenting Nesting and Breeding Ecology with Time Activity Budget of White-Throated Kingfisher (*Halcyon smyrnensis*) in Swat, Pakistan. Pak. J. Zool..

[3] Ahamed A.M.R. (2015). Activity Time Budget of the Asian Elephant (*Elephas maximus* Linn.) in the Wild. Trends Biosci..

[4] Sun Y., Li S., Li J., Wu Y., Li J. (2006). Time Budget and Activity Rhythm of Wild Great Bustard in Winter. Front. Biol. China.

[5] Watanabe S., Sato K., Ponganis P.J. (2012). Activity Time Budget during Foraging Trips of Emperor Penguins. PLoS ONE.

[6] Pépin D., Renaud P.-C., Dumont B., Decuq F. (2006). Time Budget and 24-h Temporal Rest–Activity Patterns of Captive Red Deer Hinds. Appl. Anim. Behav. Sci..

[7] Defler T.R. (1995). The Time Budget of a Group of Wild Woolly Monkeys (*Lagothrix lagotricha*). Int. J. Primatol..

[8] Huang C., Wei F., Li M., Li Y., Sun R. (2003). Sleeping Cave Selection, Activity Pattern and Time Budget of White-Headed Langurs. Int. J. Primatol..

[9] Christiansen F., Rasmussen M.H., Lusseau D. (2013). Inferring Activity Budgets in Wild Animals to Estimate the Consequences of Disturbances. Behav. Ecol..

[10] Burrows M.T., Hughes R.N. (1990). Variation in Growth and Consumption Among Individuals and Populations of Dogwhelks, Nucella Lapillus: A Link Between Foraging Behaviour and Fitness. J. Anim. Ecol..

[11] Lemon W.C. (1991). Fitness Consequences of Foraging Behaviour in the Zebra Finch. Nature.

[12] Mullers R., Navarro R. (2010). Foraging Behaviour of Cape Gannets as an Indicator of Colony Health Status. Endanger. Species Res..

[13] Walls S. (1996). Differences in Foraging Behaviour Explain Interspecific Growth Inhibition in Competing Salamanders. Anim. Behav..

[14] Aritonang S. (2009). The Effect of Forage Energy Level on Production and Reproduction Performances of Kosta Female Goat. Pak. J. Nutr..

[15] Touitou S., Heistermann M., Schülke O., Ostner J. (2021). The Effect of Reproductive State on Activity Budget, Feeding Behavior, and Urinary C-Peptide Levels in Wild Female Assamese Macaques. Behav. Ecol. Sociobiol..

[16] Inoue N., Shimada M. (2020). Comparisons of Activity Budgets, Interactions, and Social Structures in Captive and Wild Chimpanzees (*Pan troglodytes*). Animals.

[17] Ménard N., Motsch P., Delahaye A., Saintvanne A., le Flohic G., Dupé S., Vallet D., Qarro M., Pierre J.-S. (2013). Effect of Habitat Quality on the Ecological Behaviour of a Temperate-Living Primate: Time-Budget Adjustments. Primates.

[18] Kaburu S.S.K., Beisner B., Balasubramaniam K.N., Marty P.R., Bliss-Moreau E., Mohan L., Rattan S.K., Arlet M.E., Atwill E.R., McCowan B. (2019). Interactions with Humans Impose Time Constraints on Urban-Dwelling Rhesus Macaques (*Macaca mulatta*). Behaviour.

[19] Marshall H.H., Carter A.J., Rowcliffe J.M., Cowlishaw G. (2012). Linking Social Foraging Behaviour with Individual Time Budgets and Emergent Group-Level Phenomena. Anim. Behav..

[20] Canteloup C., Borgeaud C., Wubs M., van de Waal E. (2019). The Effect of Social and Ecological Factors on the Time Budget of Wild Vervet Monkeys. Ethology.

[21] Van Oers K., Naguib M. (2013). Avian Personality. Animal Personalities.

[22] Waters R.M., Bowers B.B., Burghardt G.M. (2017). Personality and Individuality in Reptile Behavior. Personality in Nonhuman Animals.

[23] Castanheira M.F., Herrera M., Costas B., Conceição L.E.C., Martins C.I.M. (2013). Can We Predict Personality in Fish? Searching for Consistency over Time and across Contexts. PLoS ONE.

[24] Kelleher S.R., Silla A.J., Byrne P.G. (2018). Animal Personality and Behavioral Syndromes in Amphibians: A Review of the Evidence, Experimental Approaches, and Implications for Conservation. Behav. Ecol. Sociobiol..

[25] Dammhahn M. (2012). Are Personality Differences in a Small Iteroparous Mammal Maintained by a Life-History Trade-Off?. Proc. R. Soc. B Biol. Sci..

[26] Stamps J., Groothuis T.G.G. (2010). The Development of Animal Personality: Relevance, Concepts and Perspectives. Biol. Rev..

[27] Réale D., Reader S.M., Sol D., McDougall P.T., Dingemanse N.J. (2007). Integrating Animal Temperament within Ecology and Evolution. Biol. Rev..

[28] Koski S.E. (2014). Broader Horizons for Animal Personality Research. Front. Ecol. Evol..

[29] Freeman H.D., Gosling S.D. (2010). Personality in Nonhuman Primates: A Review and Evaluation of Past Research. Am. J. Primatol..

[30] Moiron M., Laskowski K.L., Niemelä P.T. (2020). Individual Differences in Behaviour Explain Variation in Survival: A Meta-analysis. Ecol. Lett..

[31] Smith B.R., Blumstein D.T. (2008). Fitness Consequences of Personality: A Meta-Analysis. Behav. Ecol..

[32] Thys B., Eens M., Pinxten R., Iserbyt A. (2021). Pathways Linking Female Personality with Reproductive Success Are Trait- and Year-Specific. Behav. Ecol..

[33] Adriaenssens B., Johnsson J.I. (2011). Shy Trout Grow Faster: Exploring Links between Personality and Fitness-Related Traits in the Wild. Behav. Ecol..

[34] Biro P., Adriaenssens B., Sampson P. (2014). Individual and Sex-specific Differences in Intrinsic Growth Rate Covary with Consistent Individual Differences in Behaviour. Wiley Online Libr..

[35] Behrens J.W., von Friesen L.W., Brodin T., Ericsson P., Hirsch P.E., Persson A., Sundelin A., van Deurs M., Nilsson P.A. (2020). Personality- and Size-Related Metabolic Performance in Invasive Round Goby (*Neogobius melanostomus*). Physiol. Behav..

[36] Bergvall U.A., Schäpers A., Kjellander P., Weiss A. (2011). Personality and Foraging Decisions in Fallow Deer, Dama Dama. Anim. Behav..

[37] Kurvers R.H.J.M., Prins H.H.T., van Wieren S.E., van Oers K., Nolet B.A., Ydenberg R.C. (2010). The Effect of Personality on Social Foraging: Shy Barnacle Geese Scrounge More. Proc. R. Soc. B Biol. Sci..

[38] Andersen K.H., Marty L., Arlinghaus R. (2018). Evolution of Boldness and Life History in Response to Selective Harvesting. Can. J. Fish. Aquat. Sci..

[39] Careau V., Thomas D., Humphries M.M., Réale D. (2008). Energy Metabolism and Animal Personality. Oikos.

[40] Campos-Candela A., Palmer M., Balle S., Álvarez A., Alós J. (2019). A Mechanistic Theory of Personality-Dependent Movement Behaviour Based on Dynamic Energy Budgets. Ecol. Lett..

[41] Singh M.E., Singh M.R., Kumara H.N., Kumar M.A., D’souza L. (1997). Inter- and Intra-Specific Associations of Non-Human Primates in Anaimalai Hills, South India. Mammalia.

[42] Singh M., Kumara H.N., Ananda Kumar M., Sharma A.K. (2001). Behavioural Responses of Lion-Tailed Macaques (*Macaca silenus*) to a Changing Habitat in a Tropical Rain Forest Fragment in the Western Ghats, India. Folia Primatol..

[43] Singh M., Kumar A., Kumara H.N. (2020). *Macaca silenus*. The IUCN Red List of Threatened Species 2020: e.T12559A17951402. https://www.iucnredlist.org/species/12559/17951402.

[44] Magura T., Mizser S., Horváth R., Nagy D.D., Tóth M., Csicsek R., Lövei G.L. (2021). Are There Personality Differences between Rural vs. Urban-Living Individuals of a Specialist Ground Beetle, Carabus Convexus?. Insects.

[45] Janecka J.E., Tewes M.E., Davis I.A., Haines A.M., Caso A., Blankenship T.L., Honeycutt R.L. (2016). Genetic Differences in the Response to Landscape Fragmentation by a Habitat Generalist, the Bobcat, and a Habitat Specialist, the Ocelot. Conserv. Genet..

[46] Boydston E.E., Kapheim K.M., Watts H.E., Szykman M., Holekamp K.E. (2003). Altered Behaviour in Spotted Hyenas Associated with Increased Human Activity. Anim. Conserv. Forum.

[47] Lewis J.S., Spaulding S., Swanson H., Keeley W., Gramza A.R., Vandewoude S., Crooks K.R. (2021). Human Activity Influences Wildlife Populations and Activity Patterns: Implications for Spatial and Temporal Refuges. Ecosphere.

[48] Baruch-Mordo S., Wilson K., Lewis D., Broderick J. (2014). Stochasticity in Natural Forage Production Affects Use of Urban Areas by Black. PLoS ONE.

[49] Zeller K.A., Wattles D.W., Conlee L., DeStefano S. (2019). Black Bears Alter Movements in Response to Anthropogenic Features with Time of Day and Season. Mov. Ecol..

[50] Kifle Z., Bekele A. (2022). Time Budgets and Activity Patterns of the Southern Gelada (*Theropithecus Gelada Obscurus*) in a Human-modified Landscape, Wollo, Ethiopia. Afr. J. Ecol..

[51] Dhawale A.K., Kumar M.A., Sinha A. (2020). Changing Ecologies, Shifting Behaviours: Behavioural Responses of a Rainforest Primate, the Lion-Tailed Macaque *Macaca silenus*, to a Matrix of Anthropogenic Habitats in Southern India. PLoS ONE.

[52] Rouff J.H., Sussman R.W., Strube M.J. (2005). Personality Traits in Captive Lion-Tailed Macaques (*Macaca silenus*). Am. J. Primatol..

[53] Massen J.J.M., Antonides A., Arnold A.-M.K., Bionda T., Koski S.E. (2013). A Behavioral View on Chimpanzee Personality: Exploration Tendency, Persistence, Boldness, and Tool-Orientation Measured with Group Experiments. Am. J. Primatol..

[54] Carrier L.O., Cyr A., Anderson R.E., Walsh C.J. (2013). Exploring the Dog Park: Relationships between Social Behaviours, Personality and Cortisol in Companion Dogs. Appl. Anim. Behav. Sci..

[55] Petelle M.B., Martin J.G.A., Blumstein D.T. (2015). Heritability and Genetic Correlations of Personality Traits in a Wild Population of Yellow-bellied Marmots (*Marmota flaviventris*). J. Evol. Biol..

[56] Michelangeli M., Chapple D.G., Wong B.B.M. (2016). Are Behavioural Syndromes Sex Specific? Personality in a Widespread Lizard Species. Behav. Ecol. Sociobiol..

[57] Briffa M., Sneddon L.U. (2007). Physiological Constraints on Contest Behaviour. Funct. Ecol..

[58] Sih A., Watters J. (2005). Unravelling Animal Personalities: How and Why Individuals Consistently Differ. Behaviour.

[59] Pike T.W., Samanta M., Lindström J., Royle N.J. (2008). Behavioural Phenotype Affects Social Interactions in an Animal Network. Proc. R. Soc. B Biol. Sci..

[60] Boccia M.L., Scanlan J.M., Laudenslager M.L., Berger C.L., Hijazi A.S., Reite M.L. (1997). Juvenile Friends, Behavior, and Immune Responses to Separation in Bonnet Macaque Infants. Physiol. Behav..

[61] Altmann J. (1974). Observational Study of Behavior: Sampling Methods. Behaviour.

[62] Coss R., Ramakrishnan U. (2000). Perceptual Aspects of Leopard Recognition by Wild Bonnet Macaques (*Macaca radiata*). Behaviour.

[63] Hernández Tienda C., Beltrán Francés V., Majolo B., Romero T., Illa Maulany R., Oka Ngakan P., Amici F. (2021). Reaction to Snakes in Wild Moor Macaques (*Macaca maura*). Int. J. Primatol..

[64] Koo T.K., Li M.Y. (2016). A Guideline of Selecting and Reporting Intraclass Correlation Coefficients for Reliability Research. J. Chiropr. Med..

[65] R Development Core Team (2020). R Core Team R: A Language and Environment for Statistical Computing.

[66] Bland J.M., Altman D.G. (1997). Statistics Notes: Cronbach’s Alpha. BMJ.

[67] Budaev S.V. (2010). Using Principal Components and Factor Analysis in Animal Behaviour Research: Caveats and Guidelines. Ethology.

[68] Field A., Miles J., Field Z. (2012). Discovering Statistics Using R.

[69] Magnusson A., Skaug H., Nielsen A., Berg C., Kristensen K., Maechler M., van Bentham K., Bolker B. (2021). GlmmTMB: Generalized Linear Mixed Models Using Template Model Builder. https://cran.r-project.org/web/packages/glmmTMB/glmmTMB.pdf.

[70] Hartig F. (2020). DHARMa: Residual Diagnostics for Hierachical (Multi-Level/Mixed) Regression. https://cran.r-project.org/web/packages/DHARMa/vignettes/DHARMa.html.

[71] Hothorn T., Zeileis A., Farebrother W.R., Cummins C. (2021). Lmtest: Testing Linear Regression Models. https://cran.r-project.org/web/packages/lmtest/index.html.

[72] Revelle W. (2022). Psych: Procedures for Personality and Psychological Research. https://cran.r-project.org/web/packages/psych/index.html.

[73] Brueckl M., Heuer F. (2021). IrrNA: Coefficients of Interrater Reliability-Generalized for Randomly Incomplete Datasets. https://cran.r-project.org/web/packages/irrNA/index.html.

[74] Nijs V. (2021). Radiant.Multivariate: Multivariate Menu for Radiant: Business Analytics Using R and Shiny. https://cran.r-project.org/web/packages/radiant.multivariate/index.html.

[75] Bates D., Mächler M., Zurich E., Bolker B.M., Walker S.C. (2015). Fitting Linear Mixed-Effects Models Using Lme4. JSS J. Stat. Softw..

[76] Singmann H., Bolker B., Westfall J., Aust F., Ben-Shachar M.S. (2021). Afex: Analysis of Factorial Experiments. https://cran.r-project.org/web/packages/afex/index.html.

[77] Menon S., Poirier F.E. (1996). Lion-Tailed Macaques (*Macaca silenus*) in a Disturbed Forest Fragment: Activity Patterns and Time Budget. Int. J. Primatol..

[78] Yamanashi Y., Hayashi M. (2011). Assessing the Effects of Cognitive Experiments on the Welfare of Captive Chimpanzees (*Pan troglodytes*) by Direct Comparison of Activity Budget between Wild and Captive Chimpanzees. Am. J. Primatol..

[79] Melfi V., Feistner T.C. (2002). A Comparison of the Activity Budgets of Wild and Captive Sulawesi Crested Black Macaques (*Macaca nigra*). Anim. Welf..

[80] Jaman M.F., Huffman M.A. (2008). Enclosure Environment Affects the Activity Budgets of Captive Japanese Macaques (*Macaca fuscata*). Am. J. Primatol..

[81] Orgeldinger M. (1997). Protective and Territorial Behavior in Captive Siamangs (*Hylobates Syndactylus*). Zoo Biol..

[82] Howell C.P., Cheyne S.M. (2019). Complexities of Using Wild versus Captive Activity Budget Comparisons for Assessing Captive Primate Welfare. J. Appl. Anim. Welf. Sci..

[83] Li Y., Ma G., Zhou Q., Huang Z. (2020). Seasonal Variation in Activity Budget of Assamese Macaques in Limestone Forest of Southwest Guangxi, China. Folia Primatol..

[84] Maurice M.E., Gildas O.A.F., Ekale B.N., Fawoh J.J. (2020). The Activity Budget of Adult Chimpanzees (*Pan troglodytes* Troglodytes) and Environmental Conditions in Mefou Primate Sanctuary, Centre Region, Cameroon. Asian J. Res. Zool..

[85] Murray C.M., Lonsdorf E.V., Eberly L.E., Pusey A.E. (2009). Reproductive Energetics in Free-Living Female Chimpanzees (*Pan troglodytes Schweinfurthii*). Behav. Ecol..

[86] Uher J., Asendorpf J.B., Call J. (2008). Personality in the Behaviour of Great Apes: Temporal Stability, Cross-Situational Consistency and Coherence in Response. Anim. Behav..

[87] Tomassetti D., Caracciolo S., Manciocco A., Chiarotti F., Vitale A., de Filippis B. (2019). Personality and Lateralization in Common Marmosets (*Callithrix Jacchus*). Behav. Processes.

[88] Gartland L.A., Firth J.A., Laskowski K.L., Jeanson R., Ioannou C.C. (2022). Sociability as a Personality Trait in Animals: Methods, Causes and Consequences. Biol. Rev..

[89] Dunbar R.I. (1991). Functional Significance of Social Grooming in Primates. Folia Primatol..

[90] Arseneau-Robar T.J.M., Joyce M.M., Stead S.M., Teichroeb J.A. (2018). Proximity and Grooming Patterns Reveal Opposite-Sex Bonding in Rwenzori Angolan Colobus Monkeys (*Colobus Angolensis Ruwenzorii*). Primates.

[91] Michelangeli M., Cote J., Chapple D.G., Sih A., Brodin T., Fogarty S., Bertram M.G., Eades J., Wong B.B.M. (2020). Sex-Dependent Personality in Two Invasive Species of Mosquitofish. Biol. Invasions.

[92] Chapman B.B., Hegg A., Ljungberg P. (2013). Sex and the Syndrome: Individual and Population Consistency in Behaviour in Rock Pool Prawn *Palaemon elegans*. PLoS ONE.

[93] Schuett W., Dall S.R.X. (2009). Sex Differences, Social Context and Personality in Zebra Finches, Taeniopygia Guttata. Anim. Behav..

[94] Gartner M.C., Powell D. (2012). Personality Assessment in Snow Leopards (*Uncia uncia*). Zoo Biol..

[95] Tkaczynski P.J., Ross C., MacLarnon A., Mouna M., Majolo B., Lehmann J. (2019). Measuring Personality in the Field: An in Situ Comparison of Personality Quantification Methods in Wild Barbary Macaques (*Macaca sylvanus*). J. Comp. Psychol..

[96] Thierry B. (2007). Unity in Diversity: Lessons from Macaque Societies. Evol. Anthropol. Issues News Rev..

[97] Mitchell G., Tokunaga D.H. (1976). Sex Differences in Nonhuman Primate Grooming. Behav. Processes.

[98] Maestripieri D., Schino G., Aureli F., Troisi A. (1992). A Modest Proposal: Displacement Activities as an Indicator of Emotions in Primates. Anim. Behav..

[99] Neumann C., Agil M., Widdig A., Engelhardt A. (2013). Personality of Wild Male Crested Macaques (*Macaca nigra*). PLoS ONE.

[100] Carter A.J., Marshall H.H., Heinsohn R., Cowlishaw G. (2012). How Not to Measure Boldness: Novel Object and Antipredator Responses Are Not the Same in Wild Baboons. Anim. Behav..

[101] Pflüger L.S., Gutleb D.R., Hofer M., Fieder M., Wallner B., Steinborn R. (2016). Allelic Variation of the COMT Gene in a Despotic Primate Society: A Haplotype Is Related to Cortisol Excretion in *Macaca fuscata*. Horm. Behav..

[102] Martí O., Martí J., Armario A. (1994). Effects of Chronic Stress on Food Intake in Rats: Influence of Stressor Intensity and Duration of Daily Exposure. Physiol. Behav..

[103] Pereira A.M.F., Baccari F., Titto E.A.L., Almeida J.A.A. (2008). Effect of Thermal Stress on Physiological Parameters, Feed Intake and Plasma Thyroid Hormones Concentration in Alentejana, Mertolenga, Frisian and Limousine Cattle Breeds. Int. J. Biometeorol..

[104] Wilson M.E., Fisher J., Fischer A., Lee V., Harris R.B., Bartness T.J. (2008). Quantifying Food Intake in Socially Housed Monkeys: Social Status Effects on Caloric Consumption. Physiol. Behav..

[105] Gajdon G.K., Fijn N., Huber L. (2006). Limited Spread of Innovation in a Wild Parrot, the Kea (*Nestor Notabilis*). Anim. Cogn..

[106] Burton T., Killen S.S., Armstrong J.D., Metcalfe N.B. (2011). What Causes Intraspecific Variation in Resting Metabolic Rate and What Are Its Ecological Consequences?. Proc. R. Soc. B Biol. Sci..

[107] Biro P.A., Garland T., Beckmann C., Ujvari B., Thomas F., Post J.R. (2018). Metabolic Scope as a Proximate Constraint on Individual Behavioral Variation: Effects on Personality, Plasticity, and Predictability. Am. Nat..

[108] Thompson N.A. (2019). Understanding the Links between Social Ties and Fitness over the Life Cycle in Primates. Behaviour.

[109] Ostner J., Schülke O. (2018). Linking Sociality to Fitness in Primates: A Call for Mechanisms. Adv. Study Behav..

[110] McFarland R., Murphy D., Lusseau D., Henzi S.P., Parker J.L., Pollet T.V., Barrett L. (2017). The ‘Strength of Weak Ties’ among Female Baboons: Fitness-Related Benefits of Social Bonds. Anim. Behav..

[111] Ellis S., Snyder-Mackler N., Ruiz-Lambides A., Platt M.L., Brent L.J.N. (2019). Deconstructing Sociality: The Types of Social Connections That Predict Longevity in a Group-Living Primate. Proc. R. Soc. B.

[112] Merrick M.J., Koprowski J.L. (2017). Should We Consider Individual Behavior Differences in Applied Wildlife Conservation Studies?. Biol. Conserv..

[113] Senar J.C., Garamszegi L.Z., Tilgar V., Biard C., Moreno-Rueda G., Salmón P., Rivas J.M., Sprau P., Dingemanse N.J., Charmantier A. (2017). Urban Great Tits (Parus Major) Show Higher Distress Calling and Pecking Rates than Rural Birds across Europe. Front. Ecol. Evol..

[114] Allard S., Fuller G., Torgerson-White L., Starking M.D., Yoder-Nowak T. (2019). Personality in Zoo-Hatched Blanding’s Turtles Affects Behavior and Survival after Reintroduction into the Wild. Front. Psychol..

[115] Barlow C., Cladwell A., Lee P. Individual Differences and Response to Visitors in Zoo-Housed Diana Monkeys (*Cercopithecus Diana Diana*). Proceedings of the 8th Annual Symposium on Zoo Research.

[116] Powell D.M., Gartner M.C. (2011). Applications of Personality to the Management and Conservation of Nonhuman Animals. From Genes to Animal Behavior.

