# Supplementary material for: Personality as a Predictor of Time-Activity Budget in Lion-Tailed Macaques (Macaca silenus)
Source: Animals (Basel). 2022 Jun 8;12(12):1495. doi: 10.3390/ani12121495 (PMC9219468; doi:10.3390/ani12121495)
Supplement: Supplementary file 1 [file animals-12-01495-s001.zip › Supplementary Materials_Revised.pdf]

Table S1. Description of study animals including individual ID, housing location, date of birth, sex, parents, and rearing conditions

ID	Location	Date of Birth	Sex	Parents	Rearing Condition
Heather	Apenheul	20-07-1995	F	Tina-Homer	Parent
Sysoe	Apenheul	28-07-1998	F	Tina-Homer	Parent
Maggie	Apenheul	03-11-1999	F	Tina-Homer	Parent
Rajaja	Apenheul	12-10-2007	F	Sysoe-Billy	Parent
Salena	Apenheul	12-05-2009	F	Sysoe-Billy	Parent
Eral	Apenheul	05-11-2014	M	Parents in Ostrava	Unknown
Tumari	Apenheul	03-07-2020	M	Eral - Salena	Parent
Milo	Blijdorp	21-09-2015	M	Hera – 107056	Parent
Elly	Blijdorp	23-04-1992	F	476 - 479	Parent
Trine	Blijdorp	31-12-2016	F	Hera - 107056	Parent
Hera	Blijdorp	27-07-2008	F	107718 - 107056	Parent

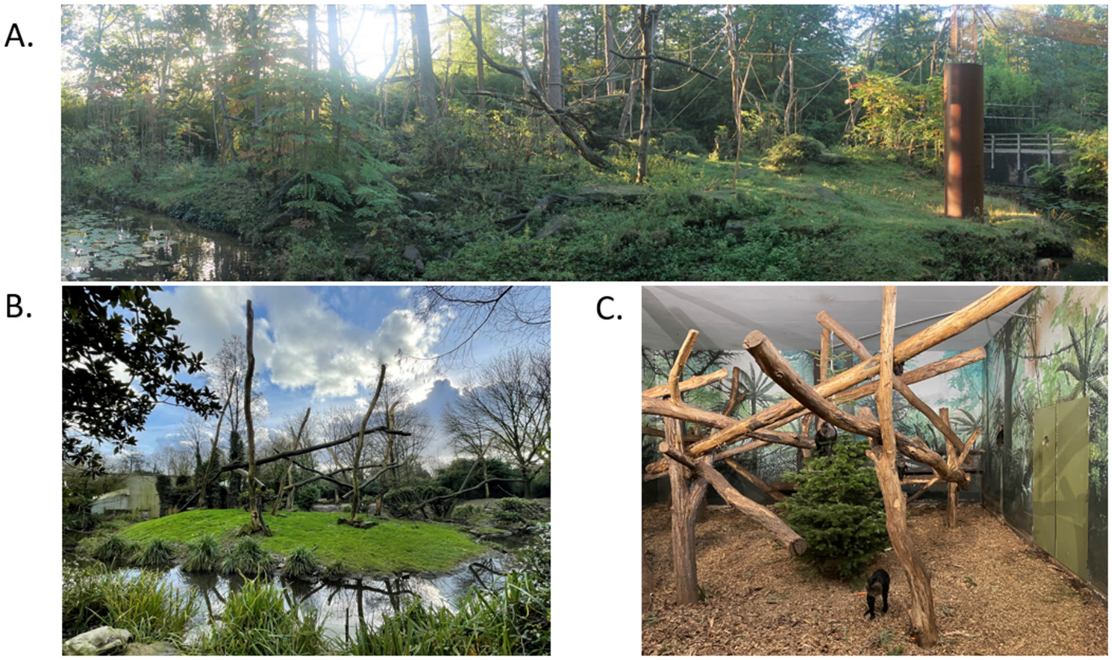

Figure S1. (a) Outdoor island at Apenheul Primate Park, Apeldoorn. Individuals were not observed inside. **(b)** Outdoor island at Blijdorp zoo, Rotterdam. **(c)** Indoor enclosure at Blijdorp zoo, Rotterdam.

Table S2. Behavioural states used to construct time-activity budgets (Adapted from Dhawale et al., 2020).

Behavioural state	Description
**Food-related**	
Drink	Individual drinks by placing mouth directly on water or by scooping water into their mouth.
Active forage	Individual actively handles or ingests food items.
Food search	Individual sits alertly and looks around in search for potential food.

Passive Feeding	Individual regurgitates food items that were stored in cheek pouches and ingests them.
Survey	Individual is alert while standing on four or two limbs or sitting. They may be focused on a specific event, group member, or object.
**Activity**	
Move	Individual moves through the enclosure on ground, branches, or platforms.
Play	Individual plays with group member(s), behaviour may consist of mock biting, chasing and lunging.
Sit alert	Individual sits while looking attentively at a specific event, group member, or object.
Autogroom	Individual grooms any part of its own body.
Stand	Individual is standing on all four limbs.
Stand alert	Individual is standing on all four limbs while focused on a specific event, group member, or object.
**Rest**	
Rest	Individual sits in a relaxed posture with eyes open.
Sleep	Individual is asleep either laying down or in a sitting position. Eyes are closed.

Table S3 Ethogram containing behaviours relating to activity, social and sexual interactions, dominance and submission, conflicts and aggression, and tension.

Behaviour	State	Description
**Activity**		
Forage	state	Focal searched for food while moving around, standing, or sitting. Focal actively eats or handles food items.
Drink	State	Focal drinks water
Regurgitation	State	Chewing of previously ingested food without actively handling food items.
Sit	State	Focal rests in posture supported by the buttocks or thigh
Travel	State	Focal walks or runs around
Stand	State	Focal stands on four legs
Bipedal stand	Event	Focal stands on hind legs
Climb	State	Focal moves in any direction on a tree, building, or construction
Hang	State	Focal hangs in place on a tree branch, rope, or other construction.
Jump	Event	Focal jumps in the air, not towards another individual
Lie down	State	Focal is lying down, either on side, belly or back
Bipedal walk	State	Focal walks standing up on both hind legs
Object play	State	Focal uses object in movements that serve no obvious, immediate purpose.
Pass by	Event	Focal enters a radius of one meter around another individual and leaves this radius within the same movement.
Coprophagy	Event	Handling and ingesting of faeces
**Social**		
Approach	Event	Focal moves towards another individual with clear focus and ends up within a three-meter radius or the focal ends up within a one-meter radius without clear focus.
Touch	Event	Focal makes gentle and short body contact with another individual
Contact sit	State	Focal is sitting next to another individual and body parts touch.
Contact lie down	State	Focal is lying next to another individual and body parts touch
Proximity	state	Focal is within one meter of another individual

Change position	Event	Focal adjusts position without leaving the immediate surroundings/the contact sit, moves less than one meter.
Co-feed	State	Focal is foraging with another individual foraging from the same source while being within one meter from each other.
Groom	State	Focal touches and strokes another individual's fur with one or both hands, accompanied by periodic hand contact with its own mouth. Focal pays close attention to the recipient's fur.
Allogrooming	State	Focal is involved in a multiple grooming interaction where the focal is being groomed and grooms at the same time
Groom present	Event	Focal approaches (or is approached by) another individual and immediately lies down when in proximity to the other individual
Social play	State	Focal displays behaviours of physical aggression (e.g., hit, grab or bite) with other individuals without vocalizations and with less intensity than regular physical aggression. Can be accompanied by play face and/or with parts of 'chasing' another individual
Embrace	State	Focal holds another individual in its arms.
Muzzle contact	Event	Focal brings its own mouth region close to another individual's mouth region.
Vocalisation	Event	Focal utters single vocalization of regular intensity and pitch (i.e., no scream, grunt, or cry for support).
Travel together	State	Focal travels parallel to another individual. Distance between the individuals is less than three meters.
Carry	State	Focal carries a juvenile on the back/belly
Temper tantrum	Event	Focal (infant) produces vocalisations in an attempt to have their mother fulfil their wishes such as clinging or embracing.
Follow	Event	Focal walks behind another individual in the same direction and stays within a radius of five meters.
Groom displace	Event	Focal displaces one monkey in a grooming dyad to form a new grooming dyad with the remaining monkey. Is always accompanied with 'groom (passive)' of the remaining monkey.
**Dominance and submission**		
Leave	Event	Focal walks away from another individual who is within a radius of one meter or after being approached by/lunged at by another individual.
Mock leave	Event	Focal walks away from another individual while turning around at least once to lunge/scream/threaten the other individual.
Displace	Event	Focal approaches another individual who then leaves, after which the focal sits within a one-meter radius where the other animal used to stay.
Lip smack	Event	Opening and closing of the lips rapidly, accompanied by smacking sound.
Avoid	Event	Focal changes its direction while walking or sitting to a distance of at least three meters of another individual that approaches the focal. Attention of the focal is on the approaching individual.
Face to face	Event	Focal pushes its own face close to the face of another individual. Usually involves grabbing the face of the other individual and pulling it towards its own.
Leave	Event	Focal walks away from another individual who is within a radius of one meter or after being approached by/lunged at by another individual.

Mock leave	Event	Focal walks away from another individual while turning around at least once to lunge/scream/threaten the other individual.
Displace	Event	Focal approaches another individual who then leaves, after which the focal sits within a one-meter radius where the other animal used to stay.
Lip smack	Event	Opening and closing of the lips rapidly, accompanied by smacking sound.
Avoid	Event	Focal changes its direction while walking or sitting to a distance of at least three meters of another individual that approaches the focal. Attention of the focal is on the approaching individual.
Face to face	Event	Focal pushes its own face close to the face of another individual. Usually involves grabbing the face of the other individual and pulling it towards its own.
**Sexual**		
Sexual mount	Event	Focal climbs on another adult individual with its hands on the receiver's back or rump. Sexual mounting might involve thrusts. Only noted when focal is in consort ship with the other individual.
Mate	Event	Focal mounts and ejaculates, which is indicated by a pause before dismounting and/or appearance of visible semen.
Masturbate	Event	Focal rubs own genitalia with fingers, on objects or the ground.
Hold bottom	Event	Focal holds another buttock or hops from behind for a short while.
Inspection	Event	Focal pulls the tail of another individual aside and visually inspects the vulva and anus.
Rejection	Event	Focal leaves or changes position with aggression while another individual shows interest in mounting.
**Conflict and aggression**		
Approach conflict	Event	Focal approaches a conflict within three meters of one involved individual. Focal does not enter the centre of the conflict and shows no involvement.
Stare	Event	Focal looks intensively at another individual with a straight back and raised eyebrows
Chase	Event	Focal runs after another individual for at least three meters.
Lunge	Event	Focal jumps a maximum of two body lengths forwards towards another individual.
Grab	Event	Focal holds body parts of another individual with one or two hands for a few seconds.
Hit	Event	Focal slaps another individual by hand.
Bite	Event	Focal places its teeth on the body of another individual and closes its jaws.
Tension		
Scratch	Event	Focal uses finger, hand or foot to rake across own skin.
Yawn	Event	Focal opens mouth wide and inhales intensely, which can be seen by the expansion of the chest.
Body shake	Event	Focal rapidly turns the whole body in at least two different directions.
Vigilance	Event	Focal has a tense body posture and looks around with hasty movements of head and/or eyes without an imminent reason. Might be accompanied by standing on the hind legs.
Focus		
Look around	Event	Focal moves its head in at least three different directions without a clear Focus. Movements are not up and down.

Attention	Event	Focal looks towards a specific situation (e.g., conflict, disturbance, or vocalization), individual or object with a clear focus without moving its head and eyes and with a frozen body posture.
**Facial expression**		
Raise brow	Event	Focal raises eyebrows at another individual, head is slightly lifted and angled. Usually displayed when an individual is approaching another.
Fear grimace	Event	Focal pulls corners of the lips back while slightly opening the mouth, teeth are visible. Often shown when receiving aggression.
Open mouth threat	Event	Focal opens his mouth for a while, directed at the receiver if aggression. Chin often points forwards.
Play face	Event	Focal opens mouth and holds that position shortly, often used to initiate play behaviour.
O-mouth	Event	Focal opens mouth as a clear 'O', not necessarily towards a conspecific. Focal is not yawning (which can follow after this movement). Lips are not pursed; it seems more like a play or movement of the mouth/lips. No teeth visible.
Affiliative bared-teeth face	Event	Focal pulls lips apart with the corners drawn backwards, mouth is partially open. Often used in a variety of non-agonistic contexts including, for example, a greeting display between adults.

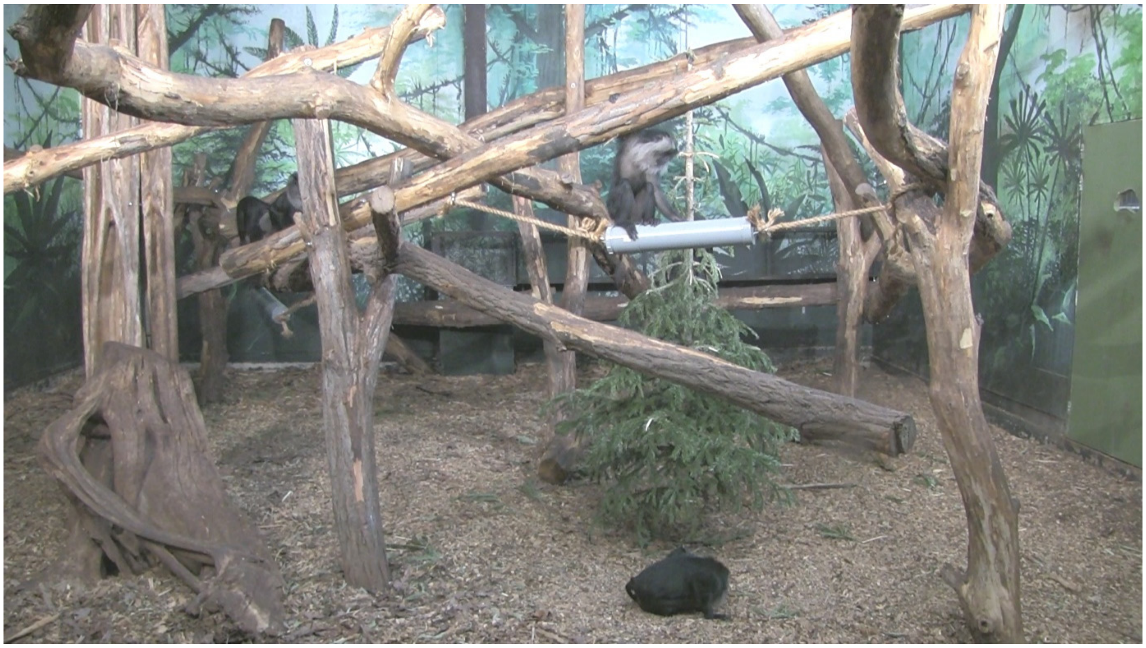

(a)

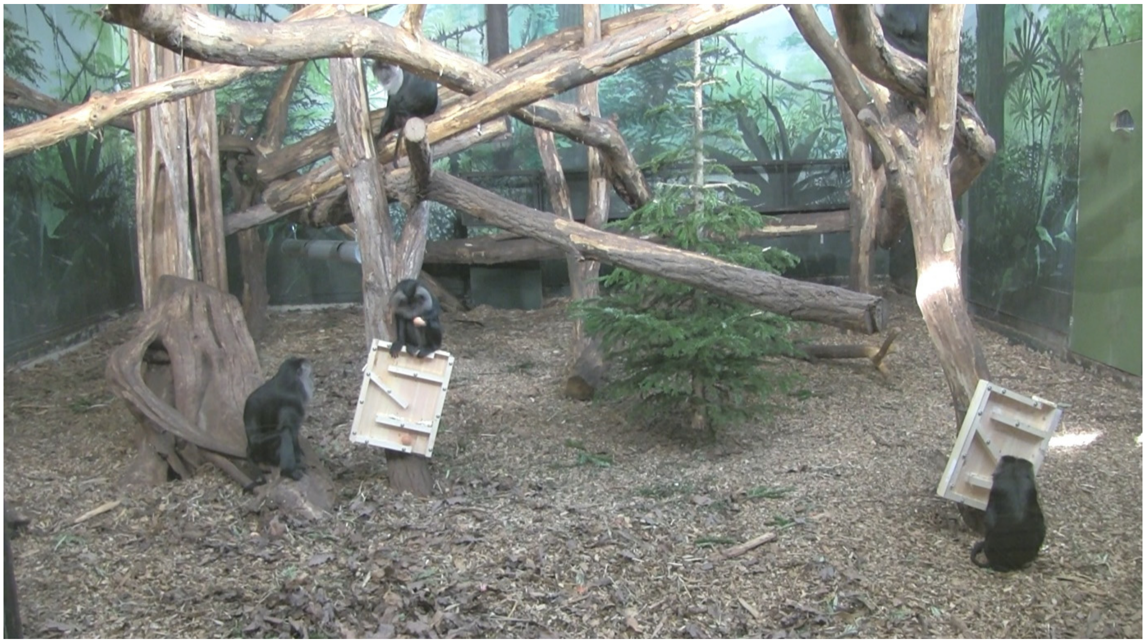

(b)

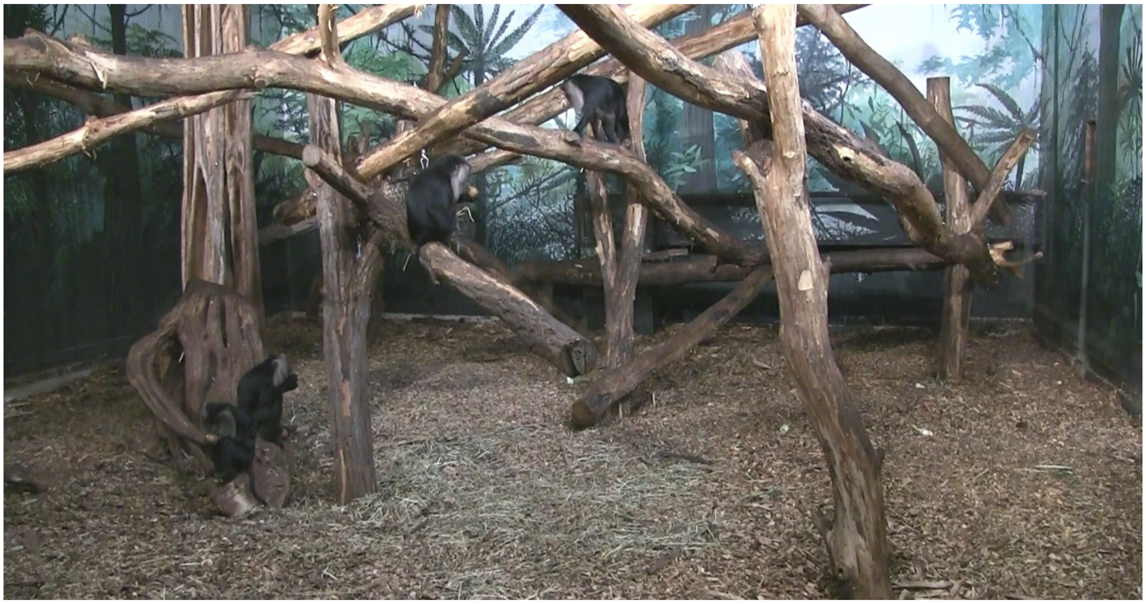

(c)

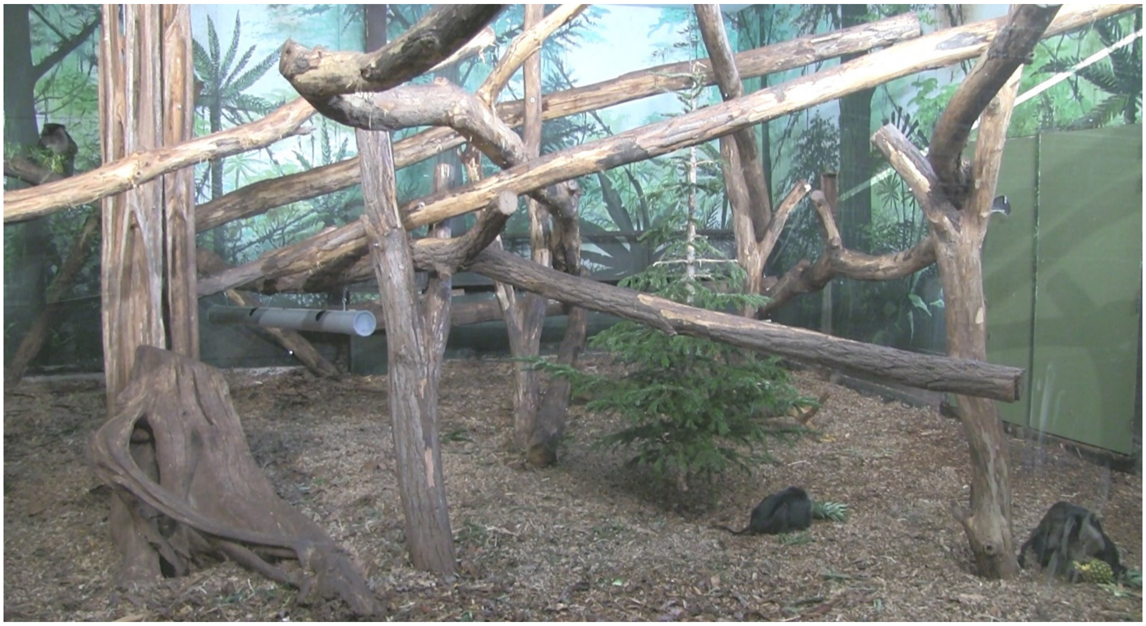

(d)

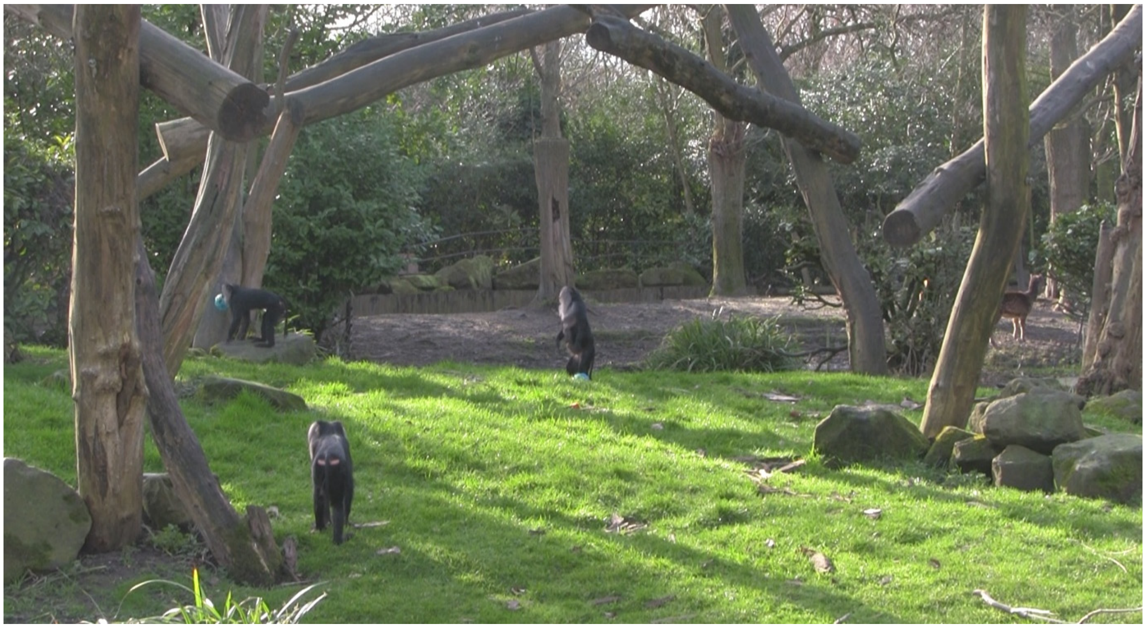

(e)

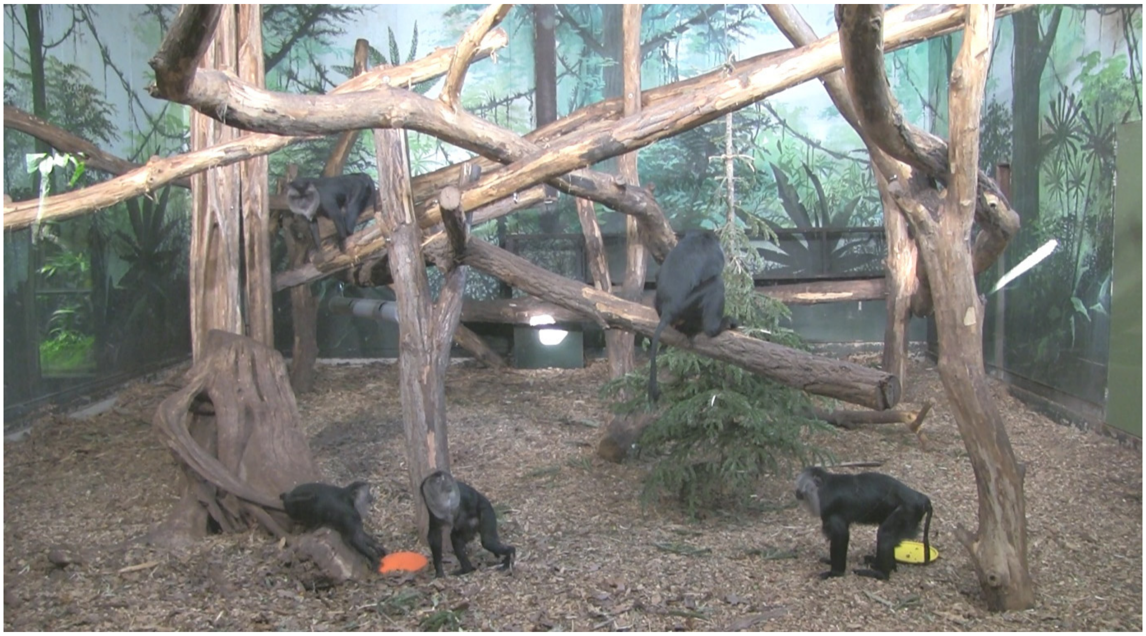

(f)

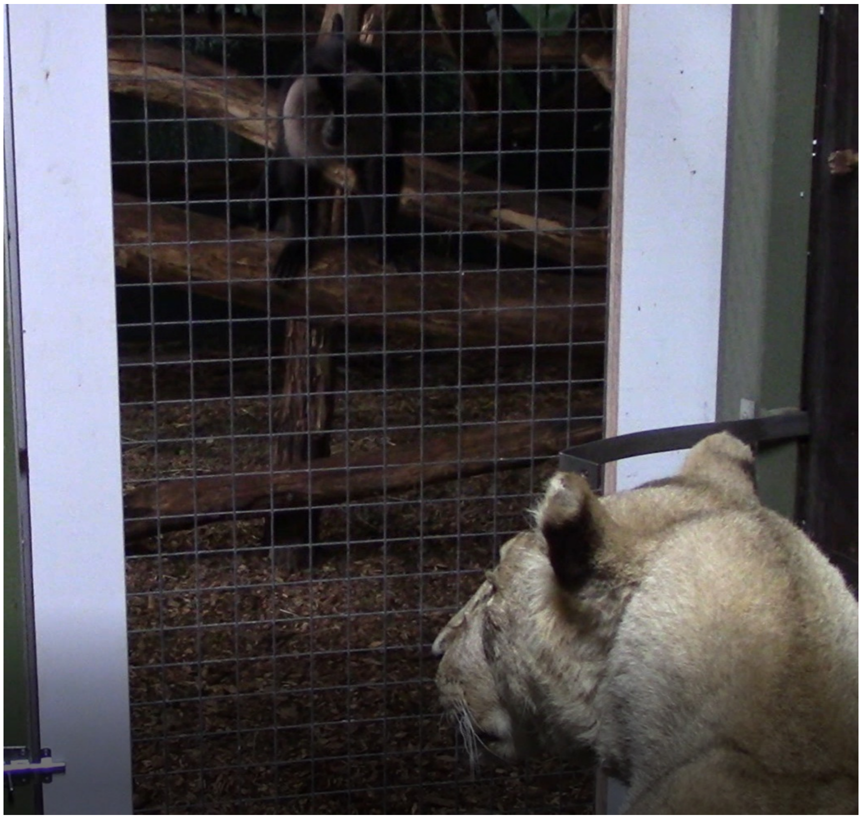

(g)

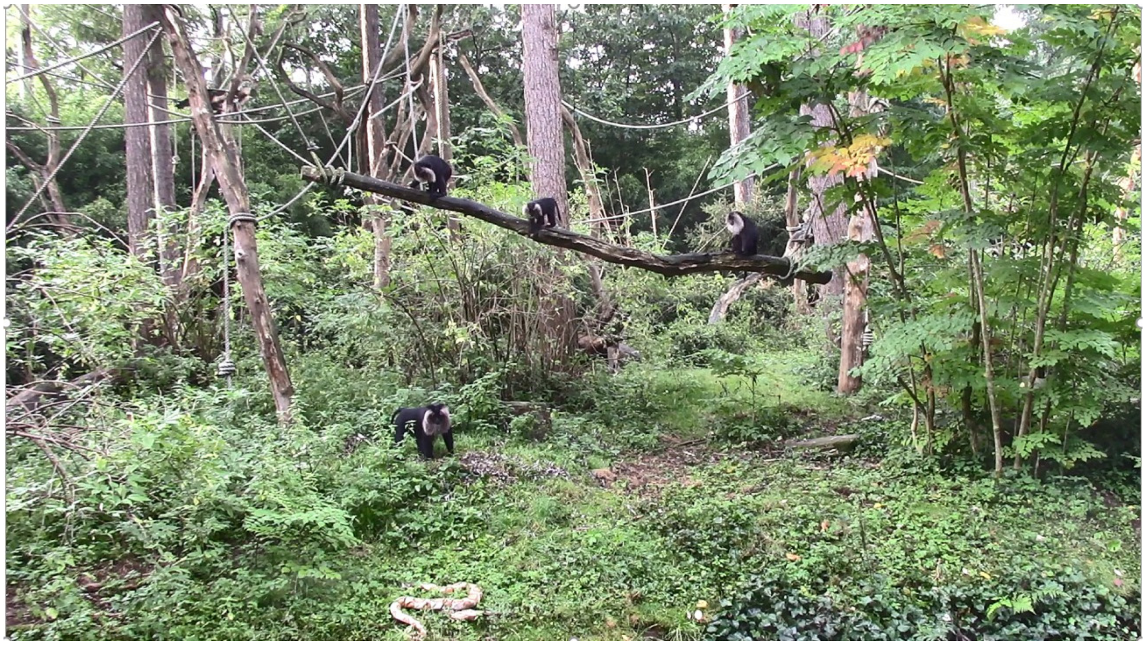

(h)

Figure S2. Personality experiments. **(a)** Food puzzle: puzzle pipes; **(b)** Food puzzle: puzzle boxes; **(c)** Novel food; kiwi; **(d)** Novel food: pineapple; **(e)** Novel object; plastic balls; **(f)** Novel object: rubber frisbee; **(g)** Predator model: Lioness; **(h)** predator model: rubber python.

Table S4. Descriptions of all measures used during novelty experiments.

Experiment	Measure	Description
Predator model	Latency to approach	Time taken by an individual to enter a two-meter radius to the predator model for the first time from an initial distance of five meters.
	Time in proximity	Cumulative time an individual spends in proximity (< two meter) to the predator model.
	Number of approaches	The total number of times an individual approaches the predator model.
Novel object	Latency to approach	Time taken by an individual to enter a one-meter radius to the predator model for the first time from an initial distance of five meters.
	Time in proximity	Cumulative time an individual spends in proximity (< one meter) to the predator model.
	Time handling	Cumulative time an individual spends handling objects.
Novel food	Latency to approach	Time taken by an individual to enter a one-meter radius to the predator model for the first time from an initial distance of five meters.
	Eat	Whether an individual eats the novel food, noted as yes or no.
Food puzzle	Latency to approach	Time taken by an individual to enter a one-meter radius to the predator model for the first time from an initial distance of five meters.
	Time in proximity	Cumulative time an individual spends in proximity (< one meter) to the predator model.
	Time manipulating	Cumulative time an individual spends manipulating food puzzles.

Table S5 Percentage of time spent on specific behavioural states (%) per individual.

	**Individuals**											
**Behaviour**	**Milo**	**Elly**	**Trine**	**Hera**	**Eral**	**Salena**	**Tumari**	**Maggie**	**Sysoe**	**Rajaja**	**Heather**	**Total**
**Food-related**												**24 ± 7**
Drink	1,38	0,57	2,51	0,56	0,41	0,00	0,00	0,00	0,87	0,00	0,00	0,57 ± 0,75
Active forage	17,13	11,11	25,07	8,36	29,22	23,53	29,41	21,77	29,13	17,77	18,36	20,99 ± 6,86
Food search	5,52	4,27	9,47	5,85	2,06	2,75	0,39	1,61	1,74	1,65	0,00	3,21 ± 2,69
Passive feeding	0,83	2,28	1,11	1,11	1,65	0,39	0,39	1,21	0,43	1,65	0,78	1,08 ± 0,57
Survey	0,28	0,00	0,28	0,00	0,00	0,39	0,00	0,00	0,00	0,00	0,00	0,09 ± 0,14

**Activity**											**21 ± 7**
Autogroom	4,70	0,85	4,46	4,74	7,41	5,88	1,18	4,03	1,74	1,24	1,56	3,43 ± 2,12
Move	19,89	14,53	19,78	11,14	17,70	12,94	14,12	11,29	14,78	8,26	12,11	14,23 ± 3,51
Play	0,28	0,00	0,28	0,00	0,41	2,35	5,49	0,00	0,43	0,83	0,39	0,95 ± 1,57
Sit-Alert	1,38	0,28	1,67	1,95	0,00	0,00	0,00	0,00	0,00	0,00	0,00	0,48 ± 0,74
Stand	6,08	1,14	1,39	4,74	4,53	3,92	2,75	1,61	0,87	0,00	1,95	2,63 ± 1,83
Stand-Alert	0,28	0,00	0,56	0	0	0	0	0	0	0	0	0,08 ± 0,17
**Resting**											**49 ± 11**
Rest	40,06	63,82	33,15	61,00	36,63	47,84	46,27	58,47	50,00	68,60	62,11	51,63 ± 11,39
Sleep	2,21	1,14	0,28	0,56	0,00	0,00	0,00	0,00	0,00	0,00	2,73	0,63 ± 0,94

Table S6. Test statistics for linearised mixed effect models testing the influence of age and sex on the multiple components. Significant results are indicated in bold typeface

Component	Measure	Estimate	Std.error	t-value	df	*p*-value
Persistence	Age	-0.061	0.035	-1.746	8	0.119
	Sex	0.381	0.709	0.537	8	0.606
Sociability	Age	-0.020	0.036	-0.551	8	0.597
	Sex	1.203	0.719	1.674	8	0.133
Affiliation	Age	-0.083	0.037	-2.258	8	0.054
	**Sex**	-1.807	0.740	-2.442	8	**0.041**
Anxiety	Age	0.046	0.047	0.982	8	0.355
	Sex	0.708	0.947	0.747	8	0.477

Table S7. Selected models for the generalised linear mixed-effect analysis with the behaviour of interests, fixed effects, random effects and corresponding one-sample Kolmogorov Smirnov test output.

Model	Behaviour of interest	Fixed effects	Random effects	One-sample Kolmogorov-Smirnov test, *p*-value	Dispersion test, *p*-value
**Personality as a predictor of time-activity budget**	Food-related behaviour	Persistence<br>Sociability<br>Affiliation<br>Anxiety	Location	0.797	0.264
	Activity	Persistence	Location	0.898	0.272

	Resting	Sociability<br>Affiliation<br>Anxiety<br>Persistence<br>Sociability<br>Affiliation<br>Anxiety	Location	0.744	0.216
**Age and sex as a predictor of time-activity budget**	Food-related behaviour	Sex	Location	0.915	0.336
	Food-related behaviour	Age	Location	0.989	0.44
	Activity	Sex<br>Age	Location	0.484	0.864
	Resting	Sex	Location	0.594	0.416
	Resting	Age	Location	0.967	0.752

Table S8. Test statistics of generalised linear mixed effect models with behavioural states as response variables, personality traits persistence, sociability, affiliation, and anxiety as fixed effects while controlling for location. Significant results are indicated in bold typeface.

**Behavioural state**	**Personality trait**	**Estimate**	**Std.error**	***z*-value**	***p*-value**
Food-related	**Persistence**	0.205	0.036	5.751	**< 0.001**
	Sociability	-0.015	0.036	-0.407	0.684
	Affiliation	-0.057	0.040	-1.414	0.157
	**Anxiety**	-0.122	0.038	-3.203	**0.001**
Activity	**Persistence**	0.219	0.040	5.589	**< 0.001**
	**Sociability**	0.131	0.039	3.379	**< 0.001**
	Affiliation	0.002	0.041	0.056	0.955
	Anxiety	0.019	0.039	0.491	0.623
Resting	**Persistence**	-0.226	0.030	-7.409	**< 0.001**
	Sociability	0.042	0.028	-1.535	0.125
	Affiliation	0.044	0.025	1.753	0.080
	Anxiety	0.021	0.027	0.787	0.431

Table S9. Test statistics for generalised linear mixed effect models with behavioural states as response variables, age and sex as fixed effects while controlling for location. Significant results are indicated in bold typeface.

**Behavioural state**	**Measure**	**Estimate**	**Std.error**	***z*-value**	***p*-value**
Food-related	**Age**	-0.015	0.008	-2.095	**0.036**
Food-related	Sex	0.182	0.172	1.061	0.289
Activity	Age	-0.017	0.010	-1.856	0.064
	Sex	0.196	0.173	0.257	0.257
Resting	**Age**	0.018	0.007	3.128	**0.002**
	**Sex**	-0.297	0.131	-2.258	**0.024**

Additional results. Results of time-activity budget analyses without the lactating female within the group.

Results are comparable to the analyses that includes the lactating female. Time spent on behavioural states differed significantly between states (Goodness of fit, $\chi2 = 468.29$, df = 2, $p < 0.001$). Individuals spent the majority of their day resting (50%), followed by food-related behavioural states (26) and activity (23%). There was no significant difference between the AP and BZ group (GLM, $z = 1.066$, $p = 0.287$). We still found variation in time-activity budget at the individual level (food-related behaviour: Goodness of fit, $\chi2 = 79.32.02$, df = 9, $p < 0.001$; activity: $\chi2 = 45.31$, df = 9, $p < 0.001$; resting: $\chi2 = 147.24$, df = 9, $p < 0.001$).

No effect of sex was found on time spent on food-related behaviour (GLMM, $z = 0.91$, $p = 0.364$), while sex was negatively correlated with time spent on food-related behaviour (GLMM, $z = -2.08$, $p = 0.036$). Furthermore, time spent being active was not predicted by sex (GLMM, $z = 0.66$, $p = 0.510$), while it is negatively correlated with age (GLMM, $z = -3.48$, $p = < 0.001$). Finally, time spent resting was predicted by sex with females resting more compared to males (GLMM, $z = -2.02$, $p = 0.044$), and also positively correlated with age (GLMM, $z = 3.96$, $p = < 0.001$).
